# The BET degrader ZBC260 suppresses stemness and tumorigenesis and promotes differentiation in triple-negative breast cancer by disrupting inflammatory signaling

**DOI:** 10.1186/s13058-023-01715-3

**Published:** 2023-11-15

**Authors:** Deeksha Sharma, Cody G. Hager, Li Shang, Lam Tran, Yongyou Zhu, Aihui Ma, Brian Magnuson, Matthew W. Lesko, Max S. Wicha, Monika L. Burness

**Affiliations:** 1 Department, Unit, and Laboratories, Rogel Cancer Center, University of Michigan, Ann Arbor, MI USA; 2 Department of Biostatistics, University of Michigan, NCRC 26-319S, SPC 2800, 2800 Plymouth Rd, Ann Arbor, MI USA; 3 Elevate Bio, Cambridge, MA USA; 4 University of Delaware, Newark, DE USA; 5 Upstate Medical University, Syracuse, NY USA

**Keywords:** Breast cancer, Cancer stem cell, BET protein degrader, ZBC260, Inflammatory cytokines

## Abstract

**Background:**

Breast cancer stem cells (BCSCs) are resistant to standard therapies, facilitate tumor dissemination, and contribute to relapse and progression. Super-enhancers are regulators of stemness, and BET proteins, which are critical for super-enhancer function, are a potential therapeutic target. Here, we investigated the effects of BET proteins on the regulation of breast cancer stemness using the pan-BET degrader ZBC260.

**Methods:**

We evaluated the effect of ZBC260 on CSCs in TNBC cell lines. We assessed the effect of ZBC260 on cellular viability and tumor growth and measured its effects on cancer stemness. We used RNA sequencing and stemness index to determine the global transcriptomic changes in CSCs and bulk cells and further validated our findings by qPCR, western blot, and ELISA.

**Results:**

ZBC260 potently inhibited TNBC growth both in vitro and in vivo. ZBC260 reduced stemness as measured by cell surface marker expression, ALDH activity, tumorsphere number, and stemness index while increasing differentiated cells. GSEA analysis indicated preferential downregulation of stemness-associated and inflammatory genes by ZBC260 in ALDH^+^ CSCs.

**Conclusions:**

The BET degrader ZBC260 is an efficient degrader of BET proteins that suppresses tumor progression and decreases CSCs through the downregulation of inflammatory genes and pathways. Our findings support the further development of BET degraders alone and in combination with other therapeutics as CSC targeting agents.

**Supplementary Information:**

The online version contains supplementary material available at 10.1186/s13058-023-01715-3.

## Introduction

Cancer stem cells (CSCs) are major drivers of breast cancer progression and therapy resistance [1]. CSCs are distinguished from the differentiated “bulk” tumor population by their capacity for self-renewal, metastasis, and drug therapy. Recent research suggests that super-enhancers (SEs) promote tumor development and resistance by inducing the expression of CSC-related genes [2]. SEs are groups of enhancers with very high levels of transcription factor binding that regulate cell-type-specific expression of genes. The bromodomain and extra-terminal domain (BET) family of proteins (BRD2, BRD3, and BRD4) are major components of the SE complex and are known to regulate the expression of many genes involved in cancer [3, 4]. Inactivation of BET proteins disrupts SE activity, thereby suppressing the expression of oncogenes such as c-MYC and other SE-associated genes [5].

Inhibition of BET proteins has been shown to decrease stemness. The BET inhibitor (BETi) JQ1 suppresses stemness by downregulating the expression of several stemness-related genes, including markers of breast CSCs (CD44, CD24, and ALDH1A1) and other important genes involved in stemness pathways [6, 7]. Another study showed that BETi treatment potently inhibits CSC self-renewal and eradicates CSCs in an in vivo mouse xenograft model [8]. However, the mechanisms by which BET proteins regulate stemness are not fully understood, and the ideal drugs to target stemness via BET proteins are not yet known.

Recently, BETi has reached clinical development, raising the possibility of regulating stemness in treating cancers. While many drugs targeting BET are small molecule inhibitors such as JQ1 [9], recent drug development has focused on targeted protein degradation. Proteolysis-targeting chimera (PROTAC) molecules are a class of targeted protein degraders that exploit the cellular ubiquitin–proteasome system to degrade a specific protein of interest, with resulting high target specificity and potency [10, 11]. Preclinical work with PROTACs against a range of targets has been promising, and two PROTAC molecules have moved into clinical trials [12, 13]. In the present study, we utilized the PROTAC pan-BET protein degrader (BETd) ZBC260 to investigate the impact of BET protein degradation on CSCs in TNBC. ZBC260 efficiently and selectively degrades BET proteins and modulates the expression of genes involved in proliferation, apoptosis, cellular viability, and tumor growth in TNBC [14, 15]. Further, our study specifically investigates the impact of BET protein degradation on cancer stem cells. Our findings demonstrate that ZBC260 effectively reduces cancer stemness and alters the CSC expression of inflammatory cytokines and chemokines..

## Materials and methods

### Survival analysis

The KM Plotter Online Tool (http://www.kmplot.com) [16], a publicly available database, was used to study the relationship between the expression of multiple BET genes (BRD2, BRD3 and BRD4) and overall survival for basal breast cancer patients. Patients were divided into two groups, high vs. low expression, based on the median value of gene expression.

### Cell culture and treatment

Cell lines SUM149, SUM159 (a gift from Dr. Stephen Ethier, Karmanos Cancer Institute, MI, the USA) (authenticated by ATCC), MDB-MB-468 and MDB-MB-453 (ATCC) were maintained in F-12 (1,765,062, Invitrogen), F-12, DMEM and RPMI 1640 medium (Cat#11,960 and 11,875, Invitrogen), respectively. Cell media were supplemented with 5% FBS, 5 µg/mL insulin, 1 µg/mL hydrocortisone, 1% Pen/strep (F12) and 10% FBS 1X antibiotic–antimycotic (DMEM and RPMI). Cells were monitored regularly for mycoplasma contamination using the MycoAlert Mycoplasma Detection Kit (Cat#LT07-318, Lonza). BETi JQ1 (Cat#A127295) was purchased from AdooQ BioScience and BETd ZBC260 was provided by Dr. Shaomeng Wang Lab, Department of Pharmacology) University of Michigan, the USA. JQ1 and ZBC260 were dissolved and diluted in DMSO and H20, respectively.

### Cellular viability and clonogenic assay

Cellular viability was measured using MTT (3-(4,5-dimethylthiazol-2-yl)-2,5-diphenyl-2H-tetrazolium bromide) assay. Briefly,1500 cells/well (SUM149) and 300 cells/well (SUM159) were plated in a 96-well low evaporation plate and cultured overnight to allow attachment followed by cells treatment with JQ1 and ZBC260 for 5 days. Cells were incubated with MTT reagent (Cat# M2128, Sigma-Aldrich) at 37 °C for 2 h. Absorbance was measured with a microplate reader at 560 nm. The clonogenic potential was measured using a clonogenic assay and crystal violet staining. Briefly, 100 cells/well were plated in 6 well plates and allowed to attach overnight prior to starting ZBC260 treatment. After 5 days of ZBC260 treatment, media was changed to new drug-free media, and the cells were allowed to grow for an additional 3 (SUM159) or 8 (SUM149) days. Cells were fixed with 100% methanol for 10 min on ice and then stained with 0.5% crystal violet (Cat# C6158, Sigma-Aldrich) in 25% methanol for 30 min on ice. Plates were imaged using the ChemiDoc-it2 imager, the number of colonies was measured using ImageJ, and the plating efficiency (PE) and survival fraction (SF) were calculated as stated below.

$${\text{PE}} = \frac{{\# \,{\text{of}}\,{\text{untreated}}\,{\text{colonies}}}}{{\# \,{\text{of}}\,{\text{cell}}\,{\text{splated}}}} \times 100$$

$${\text{SF}} = \frac{{\# \,{\text{of}}\,{\text{colonies}}\,{\text{after}}\,{\text{treatment}}}}{{\# \,{\text{of}}\,{\text{cell}}\,{\text{splated}}\, \times \,{\text{PE}}}}$$

### Western blotting

To analyze the level of protein expression, vehicle/ZBC260 treated SUM159 cells were processed for western blotting. Total protein was isolated using RIPA buffer, followed by protein quantification. A total of 30 μg of protein was run on SDS-PAGE and then transferred to PVDF membranes (Invitrogen). The PVDF membrane was blocked with BSA and then incubated overnight at 4 °C with corresponding primary antibodies (1:1000) followed by secondary antibody incubation (1:2000). The staining was detected by Super Signal West Pico Chemiluminescent substrate (ThermoFisher Scientific). Antibodies are listed in Additional file 1: Table S1.

### In vivo tumorigenesis

Female C.B.17SCID mice were purchased from Charles Rivers Laboratories and housed in pathogen-free rodent facilities at the University of Michigan. All experiments were conducted according to standards by the University Committee on the Use and Care of Animals. Briefly, SUM149 cells were injected into the fourth left inguinal mammary fat pad. After the tumors reached 200 mm3, mice were randomly divided into two groups (*n* = 10) and treated with ZBC260 (5 mg/kg) or vehicle control (20% PEG400(Cat#91,893, Sigma-Aldrich), 6% Cremophor EL (Cat#C5135, Sigma-Aldrich), three times weekly via intraperitoneal injection. Tumor size and animal weight were measured twice weekly. All animals were sacrificed when the control treatment group had tumor size reach an external measurement of 2 cm in any direction. Tumor tissue was harvested for sorting and reimplantation. Tumors were minced and digested by 1X collagenase/hyaluronidase (Cat#07912, StemCell Technologies) in medium 199 (Cat#11,150–059, Invitrogen) and filtered through a 40-mm nylon mesh. For sorting cells were pooled from multiple tumors in both the ZBC260 and vehicle treated conditions. This approach was used to ensure an “average” value for tumor initiating cell frequency. Murine cells were identified with an anti-mouse H2KD PE-conjugated antibody (Cat#116,607, BioLegend) and excluded via sorting by fluorescence-activated cell sorting. Sorted tumor cells from control and treatment groups were reimplanted at 100, 1000, and 10,000 cells and monitored for 60 days for tumor formation.

### Flow cytometry

To analyze the effect of ZBC260 on ALDH^+^ and ALDH^−^ cells population of TNBC cells we performed ALEDEFLUOR assay according to the manufacturer’s instructions. For CD24/CD44 flow cytometry, FITC Mouse Anti-Human CD24 and APC Mouse Anti-Human CD44 (BD Pharmingen™) antibodies were incubated with cells in 2% FBS/HBSS for 30 min at 4 °C. FITC and APC fluorescence were measured with a 488 nm excitation laser, a 525/40 BP fluorescence channel, and 640 nm excitation laser and 660 fluorescence channels, respectively. MoFlo® Astrios™ cell sorter (Beckman Coulter) and FlowJo software (Tree Star) were used for data acquisition and analysis, respectively.

### Sphere formation

SUM149 and SUM159 cells were plated at 20 cells and 10 cells/ well, respectively, in 96 well ultra-low attachment plates (Cat#3474, Corning) in MammoCult Basal Medium (Cat# 05621, Stemcell Technologies) with MammoCult Proliferation Supplement (Cat# 05622, Stemcell Technologies), Heparin (4ug/mL) (Cat#07980 Stemcell Technologies), and Hydrocortisone (1ug/mL) (Cat#H4001, Sigma-Aldrich). Cells were cultured for 14 days for primary spheres in the presence of ZBC260 or vehicle. For secondary sphere formation, cell clusters from primary sphere experiments were dissociated into single cells, replated, and treated as described for primary spheres. The number of spheres was assessed by counting spheres (greater than 40 µm in diameter) per well at 10 × magnification with EVOS all-in-one digital inverted microscope.

### RNA -seq

Total RNA from FACS-sorted (ALDH^+^ and ALDH^−^ cell populations) ZBC260 treated (25 nM) cells was extracted using the RNeasy Mini Kit (Cat#74,104, Qiagen), with on-column DNase treatment. RNA quality was determined using the TapeStation (Agilent) and sequencing RNA library was prepared using NEBNext Ultra II Directional RNA Library Prep Kit for Illumina (Cat#E7760L, NEB). The samples were sequenced on the Illumina NovaSeq S4 Paired end 150 bp by the University of Michigan DNA sequencing core facility. All RNA-seq reads were aligned to the human reference genome GRCh38 (ENSEMBL), using STAR v2.7.8A [17], and counts were assigned with RSEM v1.3.3 [18] followed by standardized expression values (TPM, RPKM, and FPKM).

Stemness Index (SI) was calculated by obtaining stemness weights for gene expression from a previous study [19]. We applied this stemness weight for gene expression data from the Cancer Genome Atlas (TCGA). Then, TCGA gene expression, gene expression (FPKM values from this study), and stemness weights were merged by gene symbol (11,568 genes with stemness weights) to determine the SI for our data. Differential gene expression was conducted in edge R by removing lowly expressed genes [20]. GSEA analysis was used for gene enrichment and genes and pathways were considered significantly expressed at an FDR-adjusted p value of less than 0.05 [21].

### qRT-PCR

Gene expression analysis was done in SUM159 cells sorted into ALDH^+^ and ALDH^−^ cell populations and treated with vehicle control or ZBC260 (25 nM) for 5 days. Total RNA was extracted using the RNeasy Mini Kit (Cat#74,104, Qiagen) and converted to cDNA using the Invitrogen™ SuperScript™ VILO™ cDNA Synthesis Kit (Cat#11–754-050, ThermoFisher Scientific) according to manufacturer instructions. Quantitative real-time PCR (qRT-PCR) was performed using Applied Biosystems TaqMan Gene Expression Master Mix (Cat#43–690-16, ThermoFisher Scientific) on a 7900HT Fast Real-Time PCR System (Applied Biosystems) at the Advanced genomics core facility of the University of Michigan, the USA. Primers are listed in Additional file 1: Table S2.

### Enzyme-linked immunosorbent assay (ELISA)

ELISA was performed on ALDH^+^ and ALDH^−^ cell populations of SUM159 cells treated with either vehicle control or ZBC260 (25 nM) for 5 days. After 5 days, conditioned media (CM) was collected, then centrifuged for 10 min at 1200 rpm and sterile filtered (0.2 µm) (Sigma-Aldrich) to remove debris. Cytokine determinations were performed in the Immune Monitoring Core of the Rogel Cancer Center by ELISA (Duosets, R&D Systems, Minneapolis, MN) using the manufacturer’s recommended protocol.

### Statistical analysis

Statistical analysis was performed using GraphPad Prism software 9 (San Diego, CA, the USA). Two-tailed Student t tests were used for comparing two groups and one-way and two-way ANOVA was performed for multiple group comparisons. ImageJ was used for Western blot quantification. Extreme limiting dilution analysis was performed to calculate TIC frequency [22]. A value of *P* < 0.05 was considered statistically significant.

## Results

### High BET gene expression correlates with poor survival in basal breast cancers

**Fig. 1:**
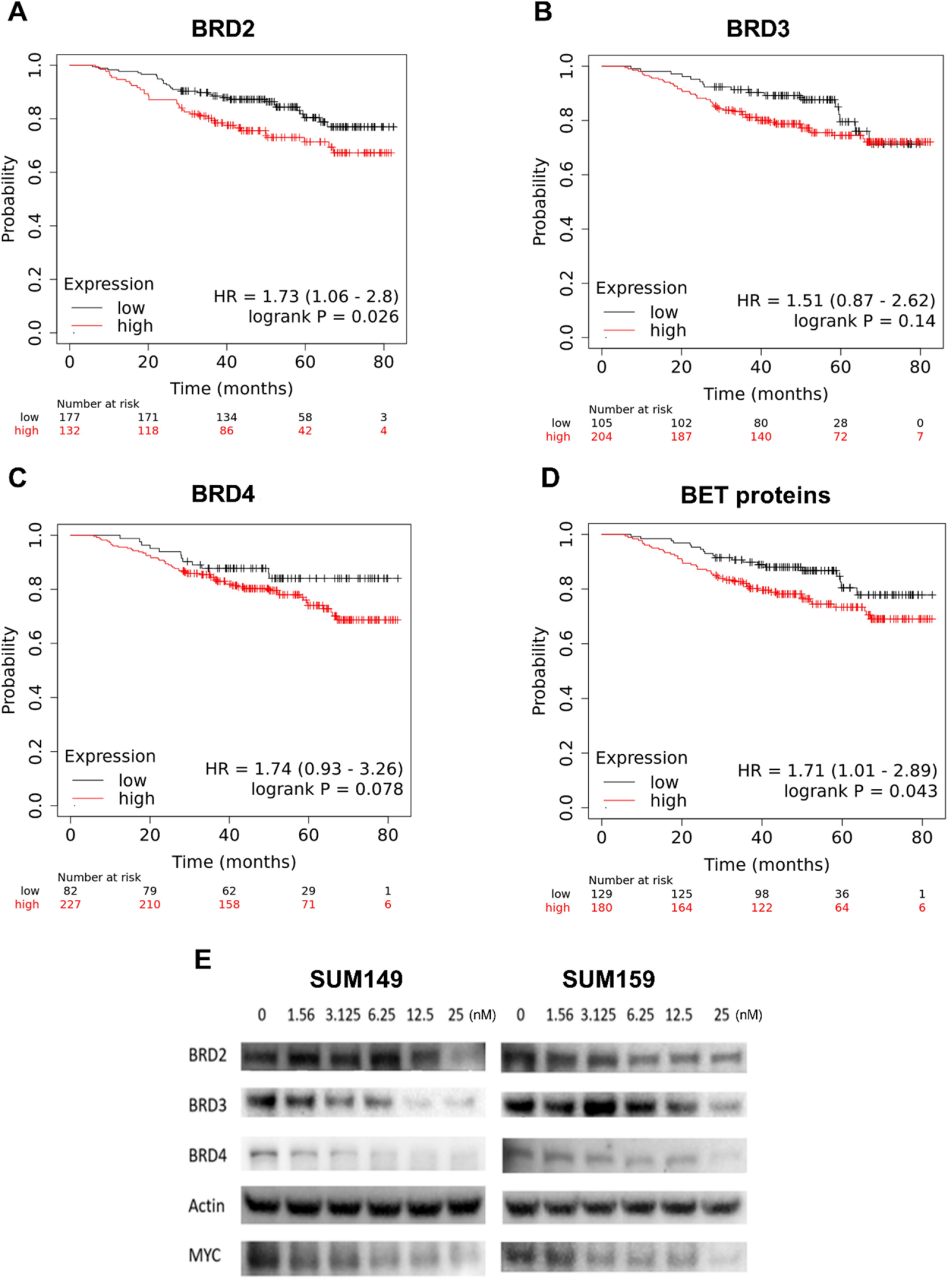
**Effect of ZBC260 on BET protein expression and TNBC cell viability**. **a**–**d** Kaplan–Meier survival curves of basal breast cancer patients based on BRD2, BRD3, BRD4, and overall expression of BET proteins. **e** Representative immunoblot of lysate from SUM149 and SUM159 cells treated with ZBC260. Protein expression was normalized to β-Actin where n = 3 independent experiments

BET proteins are involved in the transcriptional regulation of oncogenes and CSC-associated genes. To determine the prognostic relevance of BET proteins in TNBC patients, we performed Kaplan–Meier survival (KM) analysis on TCGA data of basal breast cancer patients (*n* = 309). High levels of BET gene expression were correlated with worse overall survival compared to low levels of expression (Fig. 1a–d). This correlation occurred for individual BET proteins BRD2, BRD3, and BRD4, with the greatest significance for combined BRD2, BRD3, and BRD4 expression. Therefore, we hypothesized that BET proteins are an important clinical target, and that targeted inhibition or degradation of BET proteins might improve patient outcomes. We identified the pan-BET degrader ZBC260 as a promising therapeutic agent. Consistent with previous reports [14], ZBC260 reduced breast cancer cell levels of BRD2, BRD3, and BRD4 proteins in a concentration-dependent manner. ZBC260 treatment also decreased the protein level of Myc, a gene known to be transcriptionally regulated by BRD4 (Fig. 1e and Additional file 1: Fig. S1a-b).

### ZBC260 decreases cellular viability and tumor growth

**Fig. 2:**
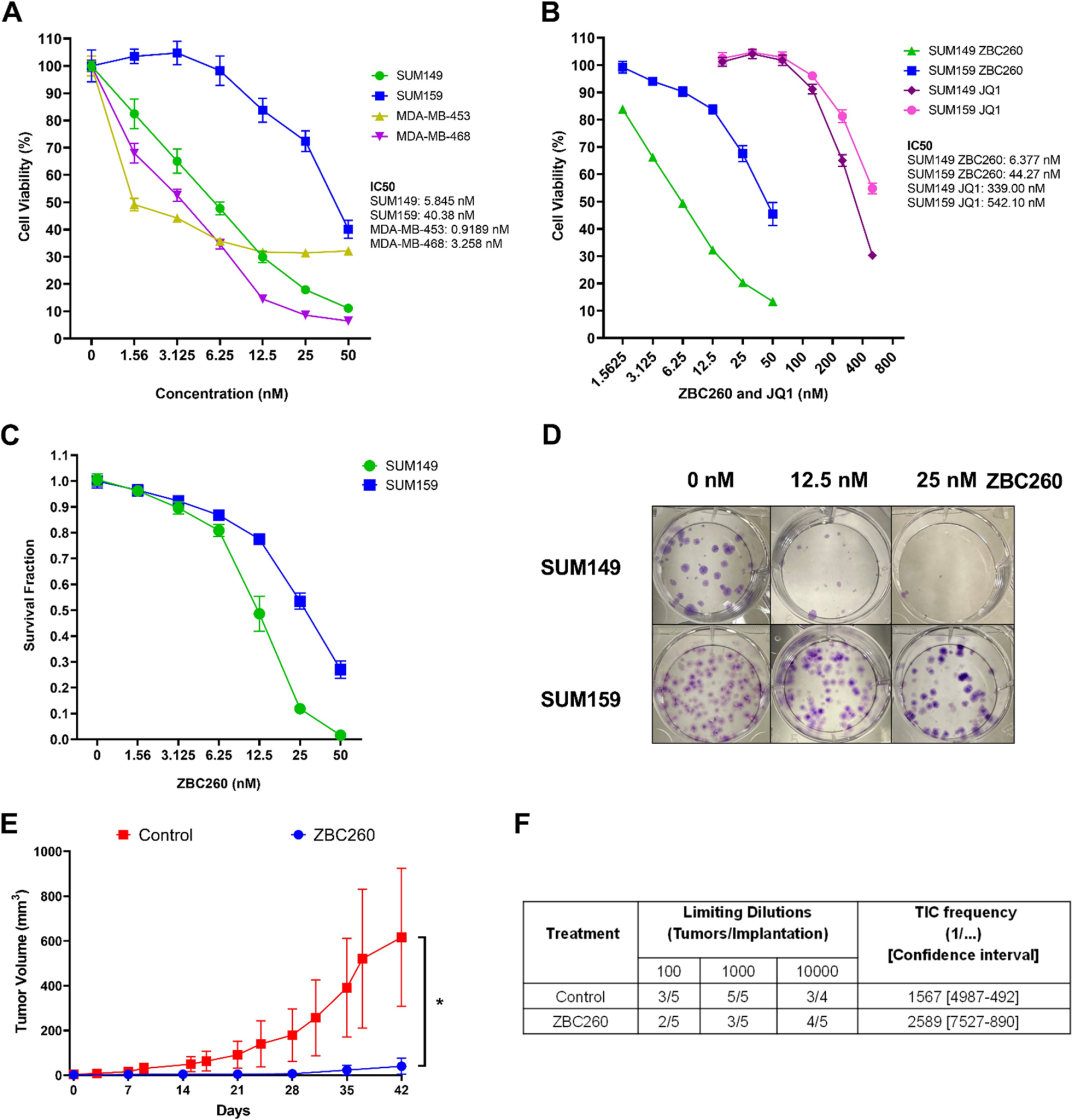
**ZBC260 significantly inhibits tumor growth in vivo**. **a** Cell viability of SUM149, SUM159, MDA-MB-453, and MDA-MB-468 cells treated with ZBC260 **b** Comparison of JQ1 vs ZBC260 on cell viability in SUM149 and SUM159 cells. The results presented are representative graphs of at least 3 biological replicates. Data points are the mean ± SEM for individual experiments. **c** Colony-formation efficiency, reported as survival fraction, of SUM149 and SUM159 cells treated with ZBC260. Mean ± SEM for *n* = 3 independent experiments. **d** Representative images of colony formation assay of SUM149 and SUM159 cells treated with 0 nM, 12.5 nM, or 25 nM ZBC260. **e** Effect of ZBC260 or vehicle on SUM149 tumor volume in C.B.17SCID mice over 42 days of treatment. **f** The tumor initiation frequency calculated by ELDA of reimplanted tumors after ZBC260 or vehicle treatment. The results presented are representative graphs of at least 3 biological replicates. Data points are the mean ± SEM for individual experiments, n = 5 independent experiments. *P < 0.05; **P < 0.01; ***P < 0.00; ****P < 0.0001

The effect of ZBC260 treatment on the cellular viability of four TNBC cell lines, SUM149, SUM159, MDA-MB-453 and MDA-MB-468, was tested in vitro. Our results demonstrated that ZBC260 treatment at nanomolar concentrations significantly decreased cellular viability in all tested cell lines (Fig. 2a), ranging from 1.56 nM (SUM149) to 12.5 nM (SUM159). To further evaluate the potency of ZBC260 compared to the well-known BET inhibitor JQ1, a comparative cell viability analysis was conducted. The results showed that ZBC260 was approximately tenfold more potent than JQ1 in inhibiting the viability of SUM149 and SUM159 cells (Fig. 2b). Additionally, the effect of ZBC260 on the ability of tumor cells to survive and form colonies, indicative of their replicative efficiency, was evaluated by colony formation assay. Treatment with ZBC260 reduced the survival fraction (SF) of cells. These findings suggest that ZBC260 not only inhibits cellular viability but also impairs the replicative efficiency of tumor cells to form colonies (Fig. 2c–d).

These in vitro results demonstrated that treatment with ZBC260 efficiently degraded BET proteins and reduced the viability of TNBC cells, thus the effect of ZBC260 on tumor growth and tumor initiation cell (TIC) frequency in vivo was determined. Mice (*n* = 10) bearing SUM149 tumor cells were treated with ZBC260 or vehicle. Compared to the control group, tumors treated with ZBC260 showed diminished tumor growth over 6 weeks of treatment (Fig. 2e, and Additional file 1: Fig. 2). Extreme limiting dilution analysis (ELDA) was performed to analyze the effect of ZBC260 treatment on TIC frequency, [22]. Tumors treated with ZBC260 had a non-significantly lower frequency of TICs compared to vehicle treated cells (Fig. 2f); coupled with the significantly decreased overall tumor size, this finding is consistent with potent drug effect against both CSCs and bulk cells.

### ZBC260 treatment decreases stemness markers, tumorsphere formation and enhances differentiation

Given the strong anti-tumor effect of ZBC260 on bulk cells and CSCs, the effect of ZBC260 on breast cancer stemness was further explored. Many CSC-targeted agents exert effects at concentrations near or even below the IC50 for the drug, and therefore, SUM149 and SUM159 cell lines were treated with ZBC260 across a range of low concentrations to assess the effect of ZBC260 on ALDH activity and CD44/CD24 expression. These two cell lines were chosen for their well-defined CSC populations: SUM149 CSCs exist as two populations defined by ALDH^+^ or CD44^+^/CD24^−^, while SUM159 CSCs are best defined by ALDH expression as most cells are CD44^+^/CD24^−^ in this very mesenchymal cell line [23].

**Fig. 3:**
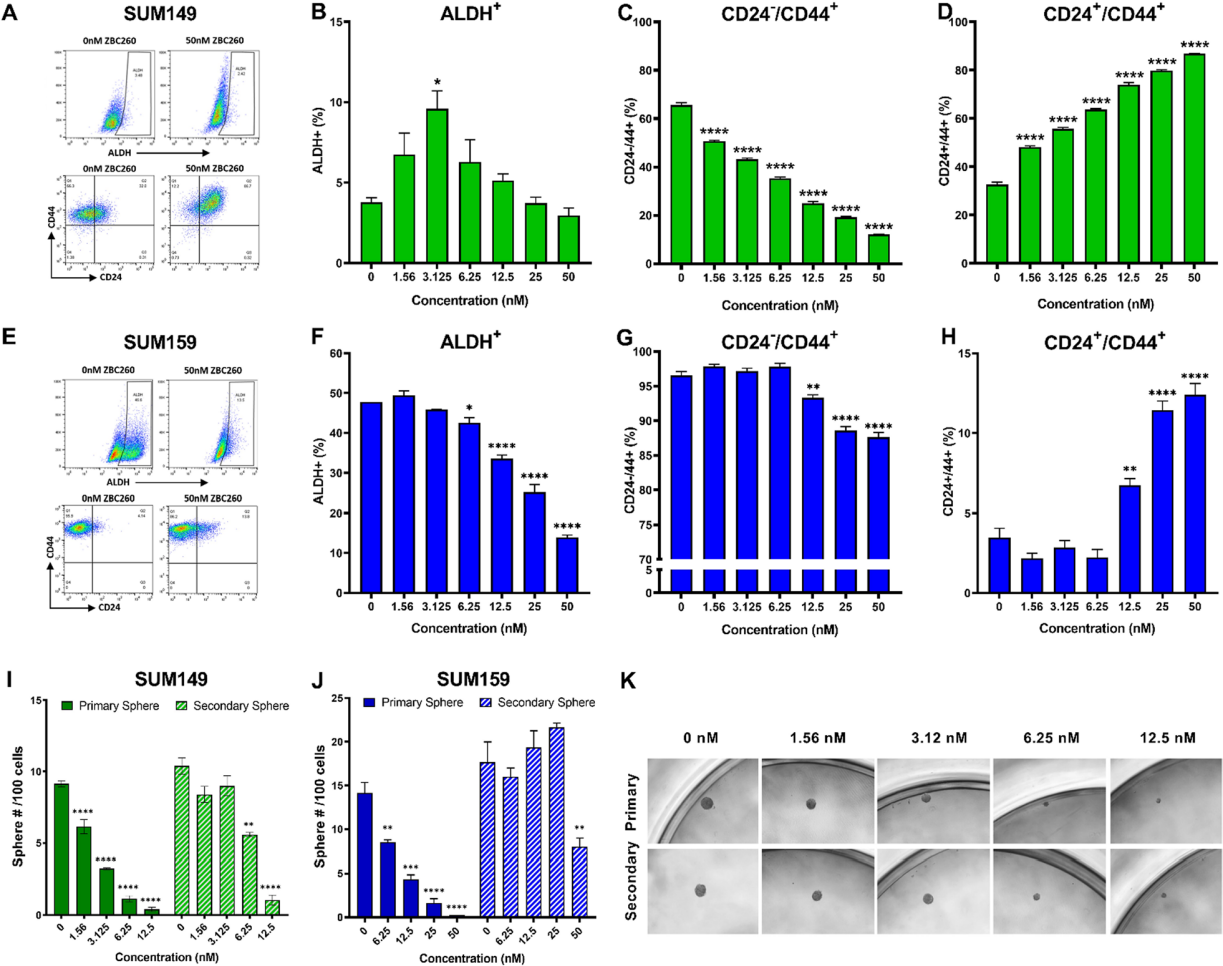
**Treatment with ZBC260 decreases markers of stemness and increases differentiation**. **a** Representative flow cytometry graphs of ALDH, CD44, and CD24 in SUM149 cells. **b**–**d** Percentage of **b** ALDH^+^ cells, **c** CD44^+^ /CD24^−^ cells, and **d** CD24^+^ /CD44^+^ cells in SUM149 cell line treated at varying concentrations of ZBC260. **e** Representative flow cytometry graphs of ALDH, CD44, and CD24 in SUM159 cells. **f**–**h** Percentage of (f) ALDH^+^ cells, **g** CD44^+^ /CD24^−^ cells, and (h) CD24^+^ /CD44^+^ cells in SUM159 cell line treated at varying concentrations of ZBC260. **i**–**j** Primary and Secondary sphere formation counts of SUM149 and SUM159 cells treated with ZBC260 at varying concentrations. **k** Size comparison of SUM159 Primary and Secondary tumorspheres. Data points are the mean ± SEM for individual experiments n = 3 independent experiments. *P < 0.05; **P < 0.01; ***P < 0.001; ****P < 0.0001

The percentage and absolute number of ALDH^+^ cells in SUM149 cells transiently increased at very low concentration of ZBC260, followed by a decrease at higher concentrations (Fig. 3a–b and Additional file 1: Fig. 3a–b), and the percentage and absolute number of CD44^+^/CD24^−^ cells in this cell line decreased in a concentration-dependent manner (Fig. 3a and c and Additional file 1: Fig. 3c). Correspondingly, the percentage of CD44^+^/CD24^+^ cells increased, inversely mirroring the observed decrease in CD44^+^/CD24^−^ cells (Fig. 3a and d).

In SUM159 cells the percentage and absolute cell number of ALDH^+^ and CD44^+^/CD24^−^ cells decreased with ZBC260 treatment (Fig. 3e–g), albeit at slightly higher concentrations compared to SUM149 cells, consistent with the higher measured IC50 in this cell line (Additional file 1: Fig. 3a, d and e). Similarly, the percentage of CD44^+^/CD24^+^ cells increased at higher concentrations (Fig. 3e and h).

The effect of ZBC260 on the absolute numbers of ALDH^+ ^and CD44^+^ /CD24^−^ cells compared to the effect on relative percentage of these cell populations indicated that both bulk cells and CSCs are sensitive to ZBC260, with the effect being more pronounced in the CSC population (Additional file 1: 4a–d).

Tumorsphere formation assays were performed to assess the effect of ZBC260 on cellular self-renewal capacity [24]. Consistent with the changes in stemness markers, we observed a concentration-dependent decrease in primary tumorsphere numbers in both cell lines. Secondary tumorsphere numbers were also decreased at higher concentrations (Fig. 3i and j). In addition to changes in tumorsphere number, the size of the tumorspheres also decreased for both primary and secondary spheres at all tested concentrations (Fig. 3k and Additional file 1: 4e–f). These results demonstrate an inhibitory effect of ZBC260 on cellular self-renewal, a key criterion for stemness.

### ZBC260 Treatment decreases stemness index and impairs the expression of stemness-related genes

**Fig. 4:**
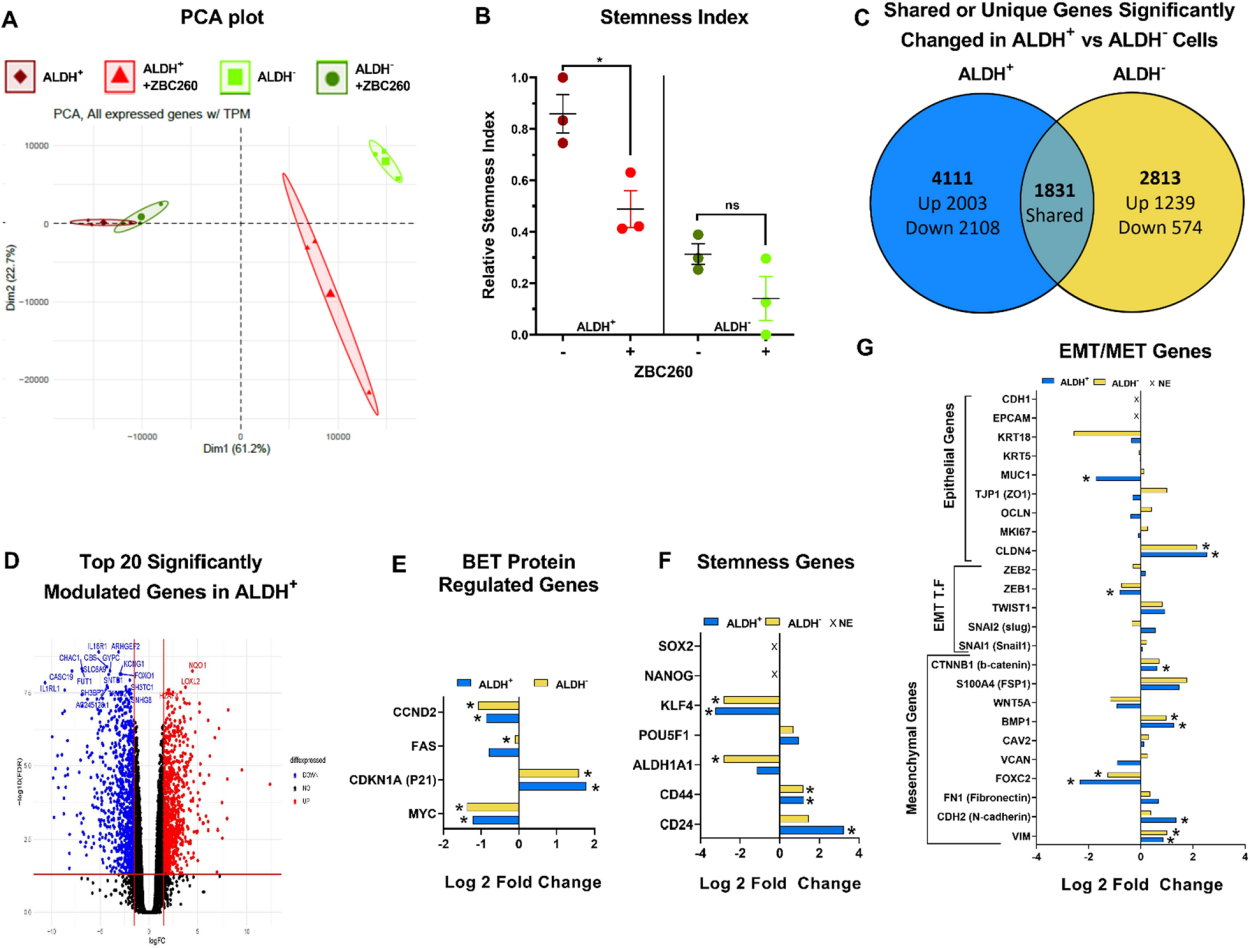
**ZBC260 modulates the expression of stemness, BET protein-regulated, and EMT-MET genes**. **a** Principal component analysis showing overall transcriptional changes in ALDH^+^ and ALDH^−^ cell populations with and without ZBC260 treatment. **b** Stemness index of ALDH and ALDH-SUM159 cells with and without ZBC260 treatment. **c**Venn diagram depicting the number of significantly differentially expressed genes to ALDH^+^ and ALDH^−^ cells population. **d** FDR Volcano plot of Top 20 significantly modulated genes in ALDH^+^ cells comparing treated to untreated cells. **e**–**g** Comparative expression of differentially expressed BET protein regulated genes, stemness genes, and EMT/MET genes in ALDH^+^ and ALDH^−^ cells after ZBC260 treatment. The legend “NE” in fig. f and g means not expressed that is SOX-2,NANOG, CDH1,& EPCAM did not expressed in RNA seq data. FDR (*P* < 0.05) was considered significant. Statistics by Benjamini–Hochberg multiple comparisons test, *n* = 3 independent experiments

To further characterize the observed changes in stemness using a molecular approach, RNA-seq was performed on SUM159 ALDH^+^ and ALDH^−^ cells treated with ZBC260 or vehicle. Principal component analysis (PCA) plot demonstrated that ALDH^+^ and ALDH^−^ populations were minimally overlapping at baseline yet become significantly distinct after ZBC260 treatment, consistent with our hypothesis that ZBC260 exerts differential effects on the ALDH^+^ and ALDH^−^ cell populations (Fig. 4a). To determine the global effect on stemness, a previously developed stemness index (SI) [19] was applied. To demonstrate the validity of applying this calculation to cultured cells, SI for treated samples was first compared to the TCGA samples originally used in the generation of the index, and the SI for our test samples fell within the range of SI values determined for TCGA samples (Additional file 1: Fig. 5a). Next, the relative SI index was determined for our 4 treatment conditions (Fig. 4b). As expected, ALDH^+^ cells had a higher SI score at baseline compared to ALDH^−^ cells. After ZBC260 treatment the SI score for ALDH^+^ population was significantly reduced (−37.11% *P* = 0.05), while ALDH^−^ cells exhibited a non-significant change in SI score (−17.26% *P* = 0.36). These findings further suggest that ZBC260 primarily affects stemness in CSCs.

Differential gene expression (DGE) analysis showed that ZBC260 treatment significantly (FDR < 0.05) modulated the expression of 4111 genes in ALDH^+^ cells and 2813 genes in ALDH^−^ cells. Of these genes, 1831 genes were shared between both cell populations, 2280 genes were unique to ALDH^+^ cells, and 982 were unique to ALDH^−^ cells (Fig. 4c). The top 20 significantly modulated genes in both cell populations were identified using unbiased gene analysis (Fig. 4d and Additional file 1: Fig. 5b). The most significantly downregulated and upregulated genes in ALDH^+^ cells were IL18R1, ARHGEF2, and CBS; and NQO1, LOXL2, and H2AFX, respectively. The corresponding genes in ALDH^−^ cells were IL18R1, ARHGEF2, and CASC19; and H2AFX, NQ01, and TUBB4B.

The expression of genes involved in three processes relevant to our investigation was analyzed: known BET-regulated genes, stemness-associated genes, and EMT/MET plasticity genes. Regarding known BET-protein-regulated genes, the BRD4 target genes MYC and CCND2 were significantly downregulated, while P21, a gene known to negatively regulate MYC expression [25], was significantly upregulated. These changes occurred in both the ALDH^+^ and ALDH^−^ populations to a similar degree (Fig. 4e). Changes in some stemness-associated genes were similar in ALDH^+^ and ALDH^−^ cells, namely downregulation of KLF4 and upregulation of CD44; however, other genes were specifically affected in only one cell population, namely CD24 which was upregulated in ALDH^+^ cells and ALDH1A1 which was downregulated in ALDH^−^ cells (Fig. 4f). We did not detect the expression of pluripotency genes SOX-2 and NANOG consistent with previous studies in this cell line [26]. Finally, the expression of genes involved in EMT-MET states was determined. Some mesenchymal genes were significantly upregulated, namely VIM, CDH2 (N-cadherin), BMP1, and CTNNB1 (b-catenin), while another mesenchymal gene FOXC2 was downregulated. Similarly, the epithelial gene CLDN4 was significantly upregulated, while MUC1 was downregulated. The EMT transcription factor ZEB1 was significantly downregulated, but no significant changes in other transcription factors were found. We observed no expression of CDH1 (E-cadherin) gene and EPCAM gene, consistent with existing characterization of SUM159 cells [27]. These results suggest minimal effects on EMT and that the effects of ZBC260 are not via modulation of the CSC EMT/MET phenotype (Fig. 4g).

### ZBC260 Suppresses inflammatory and stemness pathways

**Fig. 5:**
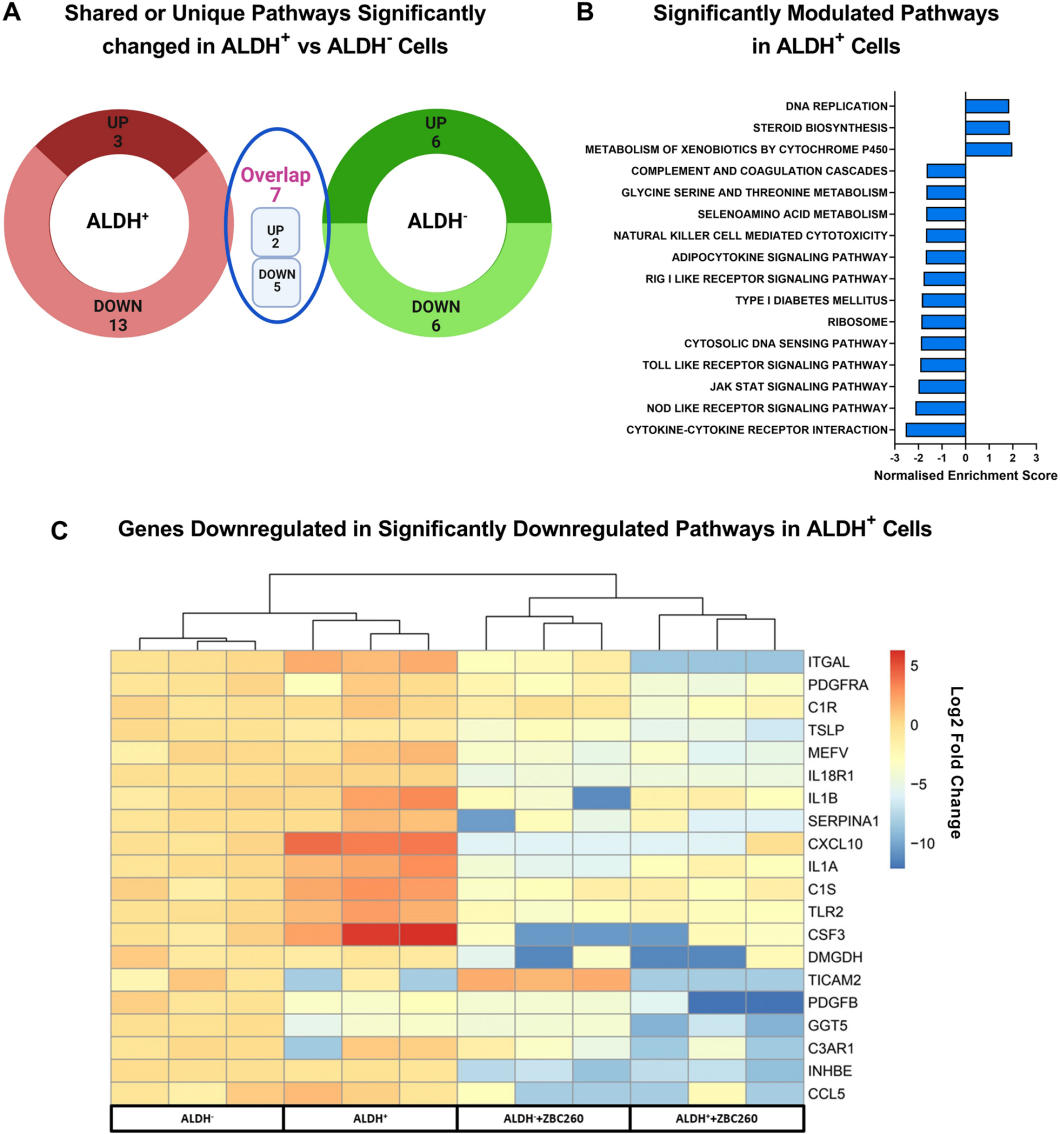
**ZBC260 modulates specific pathways in ALDH**^**+**^ **cells**. **a** Venn diagram depicting the number of significantly modulated shared or unique pathways in SUM159 ALDH^+^ and ALDH^−^ cells after ZBC260 treatment. **b** Pathways significantly regulated in ALDH^+^ and ALDH^−^ populations cells after ZBC260 treatment. **c** Top 20 significantly downregulated genes identified in significantly modulated pathways in ALDH^+^ cells after ZBC260 treatment. FDR (*P* < 0.05) was considered significant. Statistics by Benjamini–Hochberg multiple comparisons test, *n* = 3 independent experiments

These data demonstrated that ZBC260 causes significant gene expression changes in ALDH^+^ cells, with changes seen in stemness-related genes but not related to EMT/MET states. To further understand the specific effects of ZBC260 on CSCs, gene set enrichment analysis (GSEA) was performed on differentially expressed genes after ZBC260 treatment of both ALDH^+^ and ALDH^−^ cells. GSEA of KEGG pathways identified significantly (FDR < 0.05) modulated pathways in both cell types. In total 16 significant pathways were identified in ALDH^+^ cells and 12 pathways in ALDH^−^ cells; 7 pathways were shared between both cell populations, (Fig. 5a). Of the 16 significantly modulated KEGG pathways identified in ALDH^+^ cells, 3 pathways were positively enriched and 13 were negatively enriched (Fig. 5b). The cytokine–cytokine receptor interaction, NOD-like receptor signaling pathway, and JAK-STAT signaling pathway were the most significantly negatively enriched. In ALDH^−^ cells, 12 significantly regulated pathways were identified: 6 positively enriched and 6 negatively enriched (Additional file 1: Fig. 6).

To understand the drivers of the ZBC260 inhibitory effect on CSCs, the top 20 differentially expressed genes in the significantly downregulated pathways in ALDH^+^ cells were determined (Fig. 5c). Most of the identified genes were involved in inflammation, with many encoding cytokines and cytokine receptors. Top genes included IL1A, IL1B, IL18R1, PDGFRA, CSF3, INHBE, MEFV, and chemokines CCL5, CSF3, and CXCL10, all of which were reduced more than twofold after treatment in ALDH^+^ cells. Additional important genes involved in JAK-STAT signaling, including STAT4, STAT5A, and LIF, were also found to be differentially downregulated (Additional file 1: Fig. 7a, top 20 genes in the 3 most downregulated pathways). The top 20 differentially expressed genes in significantly upregulated pathways were also identified. (Additional file 1: Fig. 7b).

### ZBC260 decreased the expression of inflammatory genes and Stat proteins

**Fig. 6:**
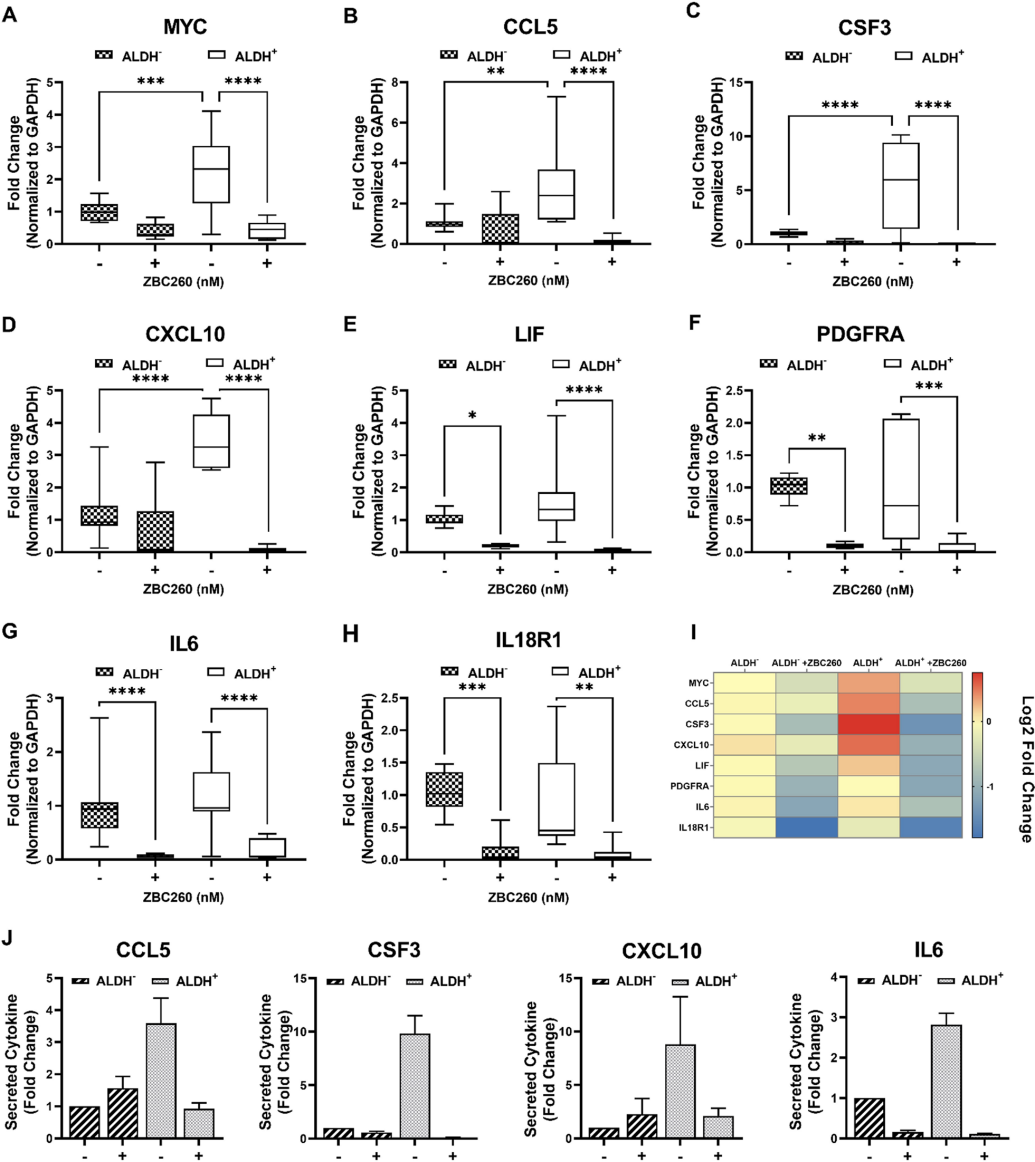
**ZBC260 decreases the expression of inflammatory genes**. (**a**–**h**) Effect of ZBC260 or vehicle treatment on gene expression in ALDH^−^ and ALDH^+^ cell populations of SUM159 cells. The expression of genes was normalized to GAPDH. **i** Heatmap showing relative RNA expression of genes after ZBC260 treatment. **j** Effect of ZBC260 or vehicle treatment on the secretion of inflammatory cytokines in ALDH^−^ and ALDH^+^ cell populations of SUM159 cells. Secretion of cytokine was measured in the conditioned media by ELISA assay and fold change was calculated relative to control. Data points are the mean ± SEM for individual experiments, *n* = 3 independent experiments. *P < 0.05; **P < 0.01; ***P < 0.001; ****P < 0.0001. The results presented are representative graphs of at least 3 biological replicates

RNA-seq data identified that the effect of ZBC260 in ALDH^+^ cells is mainly via downregulation of the expression of genes known to be involved in inflammation, potentially via JAK-STAT pathway signaling. The effect of ZBC260 on the expression of select inflammatory genes in ALDH^+^ and ALDH^−^ cells was analyzed by qPCR. These genes included the most differentially downregulated ones (CSF3, CCL5, CXCL10, IL18R1, PDGFRA), a known target gene of BRD4 (MYC), and additional genes involved in inflammatory signaling (IL-6, and LIF). ZBC260 treatment led to a significant downregulation of all the tested genes compared to control. CCL5, CSF3 and CXCL10 uniquely were more highly expressed in ALDH^+^ cells at baseline and these genes also decreased to a greater extent after ZBC260 treatment as compared to ALDH^−^ cells (Fig. 6a–i). These findings were substantiated by ELISA, showing a significant decrease in CCL5 and CSF3 secretion exclusively in ALDH^+^ cells following ZBC260 treatment, while IL6 levels decreased in both cell populations (Fig. 6j).

**Fig. 7:**
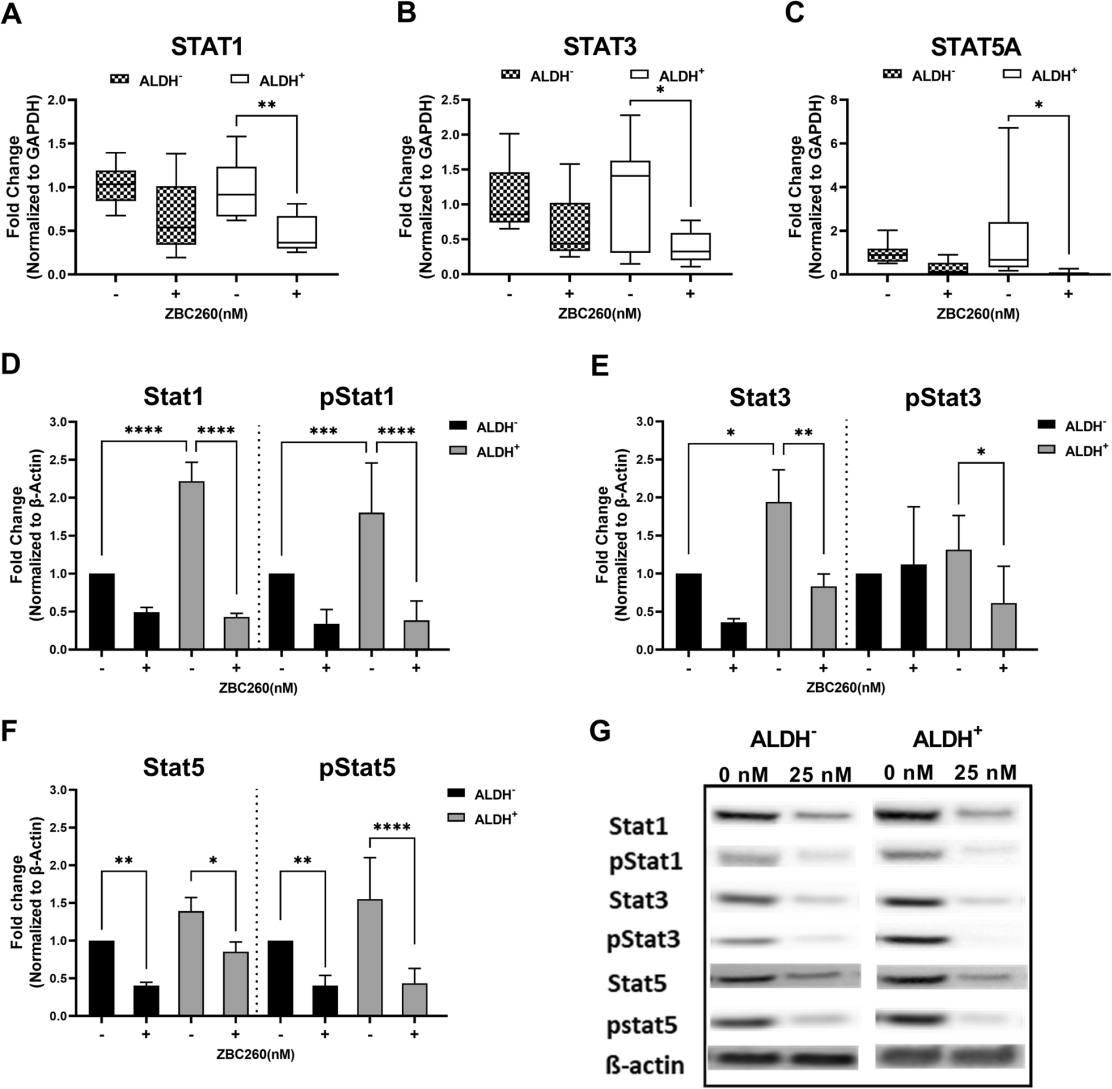
**ZBC260 decreases the expression of STAT genes and proteins**. (**a**–**c**) Effect of ZBC260 or vehicle treatment on multiple STAT gene expression in ALDH^−^ and ALDH^+^ cell populations of SUM159 cells. Expression of genes was normalized to GAPDH (**d**–**g**) Western blot analysis of Stat proteins in ALDH^−^ and ALDH^+^ cell populations of SUM159 cells treated with ZBC260. Protein expression was normalized to β-Actin. Data points are the mean ± SEM for individual experiments, *n* = 3 independent experiments. *P < 0.05; **P < 0.01; ***P < 0.001; ****P < 0.0001. The results presented are representative graphs of at least 3 biological replicates

The expression of genes that encode STAT1, STAT3, and STAT5A, was significantly decreased in ALDH^+^ cells, but not ALDH^−^ cells, after ZBC260 treatment (Fig. 7a–c). Western blot analysis showed that this decrease in gene expression led to a corresponding decrease in Stat protein levels after treatment (ZBC260) in both ALDH^+^ and ALDH^−^ populations, with a greater decrease observed in the ALDH^+^ cells compared to the ALDH^−^ cells (Fig. 7d–g). In addition to the observed decrease in total protein levels, Stat protein activation was also decreased after ZBC260 treatment as measured by phosphorylated protein levels. Levels of phosphorylated STAT3 and STAT5A were more significantly changed in the ALDH^+^ population compared to the ALDH^−^ population. These findings support a dual effect on STAT signaling in ALDH^+^ CSCs, via inhibition of both gene expression and protein activation, consistent with our RNA-seq findings which identified changes in gene expression patterns related to STAT and its signaling activators.

**Fig. 8:**
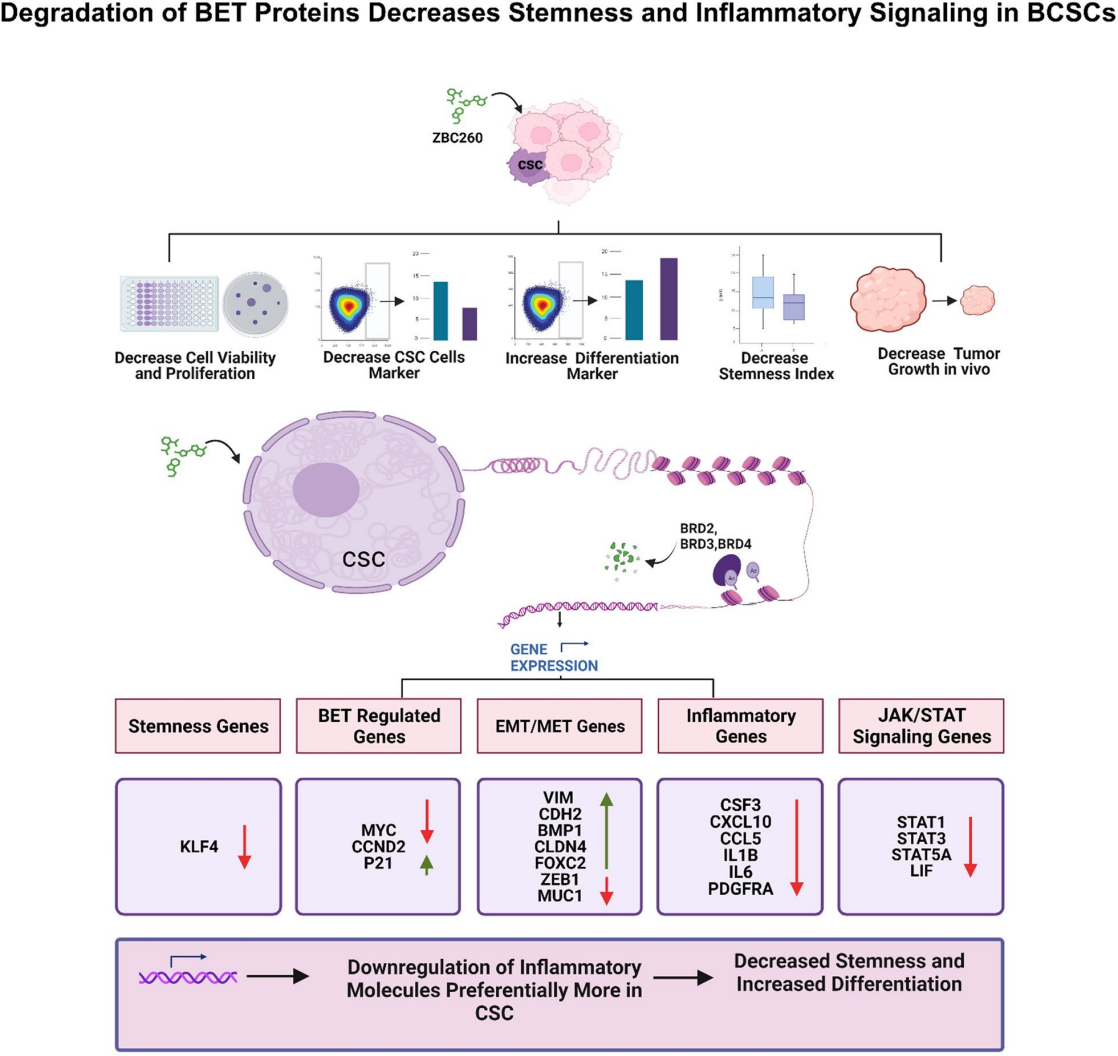
**Signaling Pathways Involved in BETd-Mediated Effects on Tumor Cell Growth and Cancer Stem Cells (CSCs)**. This figure illustrates the signaling mechanisms through which BETd (ZBC260) exerts effects on tumor cell growth and cancer stem cell (CSC) populations. BETd treatment leads to a dual impact: suppression of tumor cell proliferation and a decrease in CSC numbers. BETd mediates these effects through concentration-dependent modulation of critical molecular pathways. BETd induces differentiation and reduces stemness markers in a concentration-dependent manner, thus impairing CSC characteristics. Moreover, BETd inhibits stemness by downregulating inflammatory signaling molecules and STAT pathways. These effects are preferentially observed in CSCs, emphasizing the specificity of BETd action on CSCs

Overall, our data suggest that ZBC260 treatment not only inhibited the growth of tumor cells but also reduced the expression of CSC markers and overall stemness. ZBC260 treatment resulted in a targeted downregulation of inflammatory signaling pathways in CSCs. This concept is visually depicted in Fig. 8.

## Discussion

Recent breakthroughs in cancer research have led to a better understanding of the role of CSCs in the pathogenesis of TNBC [28] and highlighted the potential of therapeutic targeting of CSCs. Therapies based on emerging BET protein inhibitors and degraders hold promise as such CSC-targeted therapies. PROTAC degraders in general have superior selectivity, potency, and antitumor efficacy compared to small molecule inhibitors [10, 14], as demonstrated by our results with the BETd ZBC260 compared to JQ1. Consistent with previous findings, we demonstrated the potent effects of ZBC260 on cell viability in vitro [14, 29, 30]. We observed a strong anti-tumor effect in vivo that was driven by a reduction in both bulk tumor cells and CSCs. In further focusing on CSCs, we demonstrated a decrease in CSC markers, tumorsphere formation, expression of stemness genes and stemness index after ZBC260 treatment, and a corresponding increase in markers of differentiation.

Breast cancer stem cells (BCSCs) exist in two distinct states, mesenchymal (mCSC) and epithelial (eCSC). The mCSCs are identified by the CD24^−^CD44^+^ phenotype and tend to be in a quiescent state, while eCSCs express aldehyde dehydrogenase (ALDH) and display higher proliferative potential. BCSCs have the ability to transition between these two states. Previous research has demonstrated that during the differentiation of mCSCs into bulk cells, they initially shift toward an eCSC phenotype before fully differentiating [31]. Our findings in SUM149 cells of a transient increase in ALDH^+^ cells at lower concentrations of ZBC260 followed by a subsequent decrease at higher concentrations coupled with a decrease in CD44^+^/CD44^−^ across all concentrations may be consistent with such a transition [32–34]. A reduction in the ALDH^+^ cells at higher concentrations of ZBC260 can potentially be attributed to the increasing cytotoxic effects of the compound, which outweigh its differentiation potential. Overall, results of multiple measures of stemness indicate that the pan-BETd ZBC260 can effectively decrease the population of CSCs by inducing their transition toward a more differentiated state [35].

To understand the key regulatory pathways driving the effect of ZBC260 on CSCs, we performed GSEA on ALDH^+^ and ALDH^−^ CSCs. By analyzing changes induced by ZBC260 in ALDH^+^ and ALDH^−^ cells, we identified CSC-specific effects of BET protein degradation. Our results identified significant downregulation of inflammatory pathways including cytokine signaling and NOD-like receptor in both cell populations. Furthermore, the JAK-STAT pathway, another inflammatory pathway, was specifically downregulated in ALDH^+^ CSCs. These findings are consistent with previous studies demonstrating the downregulation of inflammatory genes by JQ1, a related BET protein inhibitor [36]. Inflammation is a hallmark of cancer and critical for the maintenance of CSCs [37, 38]. The relationship between CSCs and the inflammatory microenvironment is regulated via an intricate, balanced network of mediators [39] and signaling pathways [40–42]. Our finding that BETd ZBC260 decreases CSCs by altering inflammatory signaling supports a key link between BET proteins and CSCs. The preferential downregulation of JAK-STAT signaling in ALDH^+^ cells is a noteworthy effect of ZBC260. STAT pathway is known to be a critical regulator of CSCs [41, 42]. Our study identified CSC-specific gene expression changes related to JAK/STAT signaling, including genes encoding proteins upstream and downstream of STAT pathway and several STAT genes. We specifically identified key inflammatory genes, including CSF3, and CCL5, that are differentially downregulated in ALDH^+^ cells after ZBC260 treatment. These observed genes are involved in the positive crosstalk between CSCs and the tumor immune microenvironment and aid in maintaining the CSC niche within the tumor microenvironment [43, 44] by modulating JAK-STAT signaling [41, 45, 46].

Consistent with these findings, we demonstrated that BETd ZBC260 decreased STAT protein activation. These data align with other studies showing that BETi decreases JAK/STAT signaling primarily via the downregulation of inflammatory cytokines [47]. Our novel findings of differential regulation of these genes in ALDH^+^ CSCs compared to bulk tumor cells provide a potential mechanism for the CSC targeting effects of ZBC260. Furthermore, we observed that BET degradation led to a significant decrease in the expression of STAT1, STAT3, and STAT5 at both the protein and mRNA levels, a new finding which warrants further exploration in future studies. Thus, our data provide two complementary mechanisms by which BET degradation leads to the downregulation of JAK-STAT signaling preferentially in ALDH^+^ cells. Our unbiased analysis of key genes and pathways suggests that ZBC260 negatively regulates breast cancer stemness by suppressing key inflammatory molecules and STAT genes, associated with the JAK-STAT signaling pathway known to be involved in driving the cancer stem cell (CSC) phenotype. Further investigations are required to establish a causative link between ZBC260 effects on JAK-STAT signaling and CSC regulation, and to elucidate the specific molecular mechanisms underlying the effect of ZBC260 on CSCs.

In addition to regulating key CSC signaling pathways in tumors, BET proteins also regulate immune function. Our data demonstrate that BET degradation leads to significant changes in inflammatory signaling in CSCs. While our studies primarily explored the direct effect of ZBC260 on tumor cells, given the important role of inflammatory signaling in the tumor microenvironment and the immune system, our work has important implications within the context of the tumor immune microenvironment. An important limitation of this study is that we have not yet investigated the role of the immune system in the effect of ZBC260 in vivo. However, several BET proteins are known to govern immune cell functions [48] and regulate the secretion of immune cytokines by modulating immune cell interactions [49]. Conversely, BET inhibition has demonstrated the potential to enhance anti-tumor immunity [50–52] by influencing the expression of immune-related genes [53], enhancing NK cell-mediated cytotoxicity [50], and improving responses to immune checkpoint therapy, both as a monotherapy [54] and in combination with other immunotherapeutic agents [55]. These findings suggest that use of a pan-BET degrader, as opposed to a more specific BRD4 specific drug, may be optimal for immune regulation. Future studies are warranted on the interplay of CSCs and immune cells in the presence of BET targeting drugs, and our findings thus far suggest potential therapeutic efficacy from ZBC260 due to immune modulation that may synergize with immune checkpoint inhibition or other immunotherapeutics.

## Conclusions

In conclusion, we demonstrated that the pan-BETd ZBC260 decreases TNBC stemness while promoting differentiation. Our finding suggests that this key shift in stemness is due to the disruption of key inflammatory signaling and via modulation of multiple aspects of the JAK/STAT pathway. Therapies that target CSCs by driving differentiation can sensitize CSCs to conventional treatments by converting them to more therapy-sensitive cells. Use of BET protein regulation to modulate inflammation via STAT signaling represents a promising CSC-targeted therapeutic.

### Supplementary Information

**Additional file 1**. Supplementary data supporting main figures. **Supplementary Figure 1**. Effect of ZBC260 treatment on BET and Myc proteins expression. **Supplementary Figure 2**. Representative tumor growth picture at the end of the experiment. **Supplementary Figure 3**. Effect of ZBC260 treatment on the absolute number of ALDH^+^ and CD44^+^/CD24^−^ cells. **Supplementary Figure 4**. Effect of ZBC260 treatment on ALDH^+^ and CD44^+^/CD24^−^ marker of bulk cells and CSC cells and tumorsphere size. **Supplementary Figure 5**. Effect of ZBC260 treatment on the transcriptome of SUM159 cells. **Supplementary Figure 6**. Effect of ZBC260 treatment on signaling pathways in ALDH^−^ cells. **Supplementary Figure 7**. Effect of ZBC260 on signaling pathways in ALDH^+^ cells. **Supplementary Table 1**. Antibodies used for Western Blotting. **Supplementary Table 2**. Human TaqMan Gene Expression Assay primer/probes (Thermo Fisher Scientific) used for real-time quantitative PCR.

**Additional file 2**. Uncropped western blots relating to manuscript figures. (**a**–**e**) Uncropped western blots for (**a**) BRD2, (**b**) BRD3, (**c**) BRD4, (**d**) Actin, and (**e**) MYC; as shown in Figure 1. Lane 1 & 15 – ladder (198-3kDa), lanes 2-7 – SUM149 treated with 0, 1.56, 3.125, 6.25, 12.5, and 25 nM ZBC260 respectively, lane 8 – ladder (460-31kda), and lanes 9-14 – SUM159 treated with 0, 1.56, 3.125, 6.25, 12.5, and 25 nM ZBC260 respectively. (**f**–**l**) Uncropped western blots of ALDH^−^ and ALDH^+^ SUM159 cell treated with ZBC260 (**f**) Stat1, (**g**) pStat1, (**h**) Stat3, (**i**) pStat3, (**j**) Stat5, (**k**) pStat5, and (**l**) β-actin; as shown in Figure 7. Lane 1 & 15 – ladder (198-3kDa), lanes 2-4 – SUM159 ALDH^−^ + Control, lanes 5-7 – SUM159 ALDH^−^ + ZBC260, lane8 – ladder (460-31kda), lanes 9-11 – SUM159 ALDH^+^ + Control, lanes 5-7 – SUM159 ALDH^+^ + ZBC260.

## Data Availability

Some raw data for this study were generated at Flow Core and Advanced Genomics Core of the University of Michigan, the USA. Derived data supporting the findings of this study are available from the corresponding author upon request.

## Abbreviations

ALDH: Aldehyde dehydrogenase
APC: Allophycocyanin
BET: Bromodomain and extra-terminal domain
BETd: BET protein degrader
BETi: BET protein inhibitor
CSC: Cancer stem cell
EMT: Epithelial-mesenchymal transition
FDR: False discovery rate
FITC: Fluorescein isothiocyanate
GSEA: Gene set enrichment analysis
JAK/STAT: Janus kinase/signal transducers and activators of transcription
KEGG: Kyoto Encyclopedia of Genes and Genomes
MET: Mesenchymal-epithelial transition
NOD: Nucleotide-binding oligomerization domain
PCA: Principal component analysis
PD-L1: Programmed death ligand 1
PROTAC: Proteolysis targeting chimera
RPKM: Reads per kilobase of transcript per million reads mapped
SE: Super-enhancer
STR: Short tandem repeat
TAM: Tumor-associated macrophage
TCGA: The Cancer Genome Atlas
TNBC: Triple-negative breast cancer
TPM: Transcript per million

## Availability of data and materials

The data supporting the findings presented in this study are available upon reasonable request from the corresponding author, M.B.
