# Supplementary material for: The BET degrader ZBC260 suppresses stemness and tumorigenesis and promotes differentiation in triple-negative breast cancer by disrupting inflammatory signaling
Source: Breast Cancer Res. 2023 Nov 15;25:144. doi: 10.1186/s13058-023-01715-3 (PMC10648675; doi:10.1186/s13058-023-01715-3)
Supplement: Supplementary file 1 — Additional file 1. Supplementary data supporting main figures. Supplementary Figure 1. Effect of ZBC260 treatment on BET and Myc proteins expression. Supplementary Figure 2. Representative tumor growth picture at the end of the experiment. Supplementary Figure 3. Effect of ZBC260 treatment on the absolute number of ALDH+ and CD44+/CD24− cells. Supplementary Figure 4. Effect of ZBC260 treatment on ALDH+ and CD44+/CD24− marker of bulk cells and CSC cells and tumorsphere size. Supplementary Figure 5. Effect of ZBC260 treatment on the transcriptome of SUM159 cells. Supplementary Figure 6. Effect of ZBC260 treatment on signaling pathways in ALDH− cells. Supplementary Figure 7. Effect of ZBC260 on signaling pathways in ALDH+ cells. Supplementary Table 1. Antibodies used for Western Blotting. Supplementary Table 2. Human TaqMan Gene Expression Assay primer/probes (Thermo Fisher Scientific) used for real-time quantitative PCR. [file 13058_2023_1715_MOESM1_ESM.pdf]

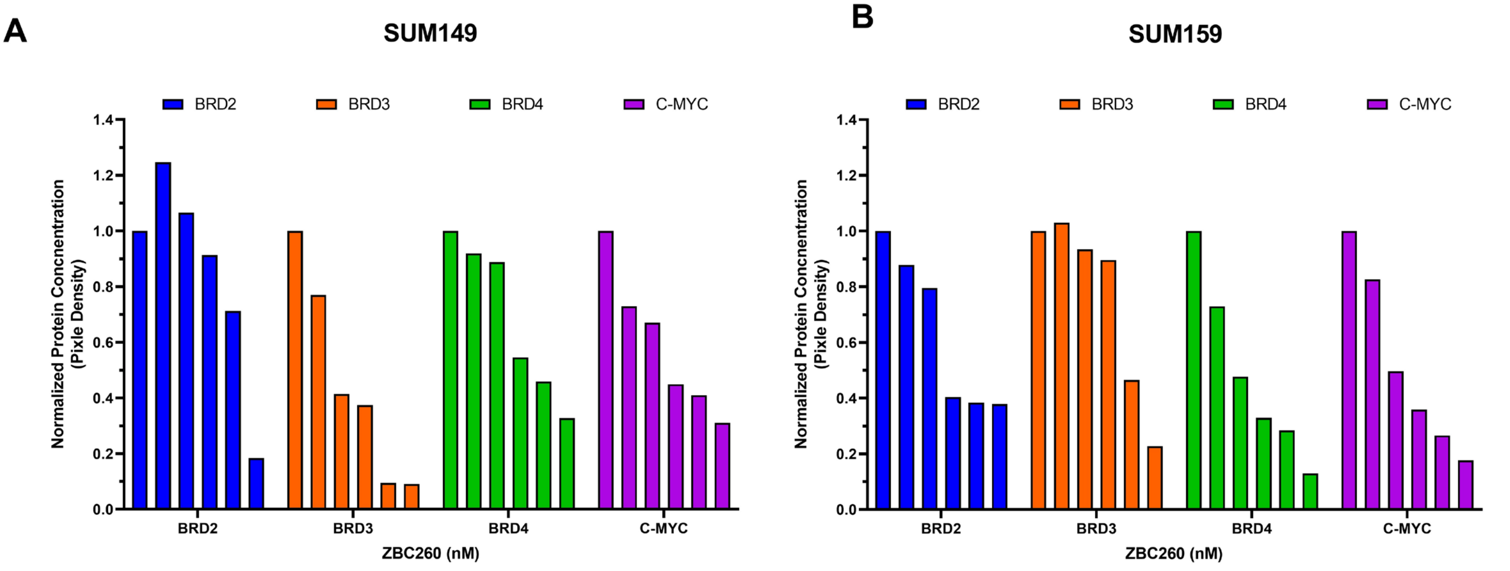

Supplementary Figure 1. Effect of ZBC260 treatment on BET and Myc proteins expression.
(a-b) Concentration-dependent effect of ZBC260 on the expression of BRD2, BRD3, BRD4, and C-MYC protein in SUM149 cells (a) and SUM159 cells (b). Data points are the mean ±SEM for individual experiments, n= 3 independent experiments.

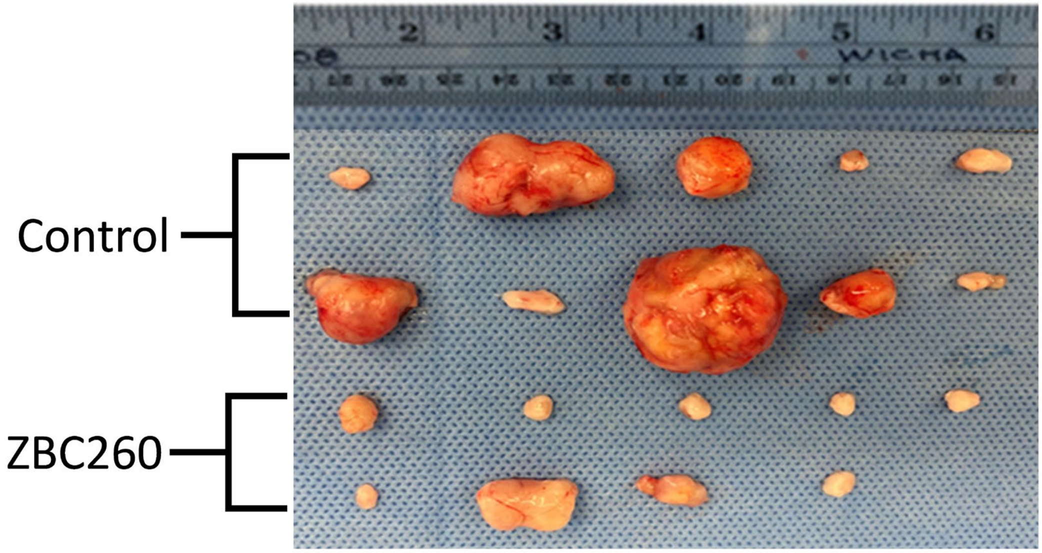

Supplementary Figure 2. Representative tumor growth picture at the end of the experiment.

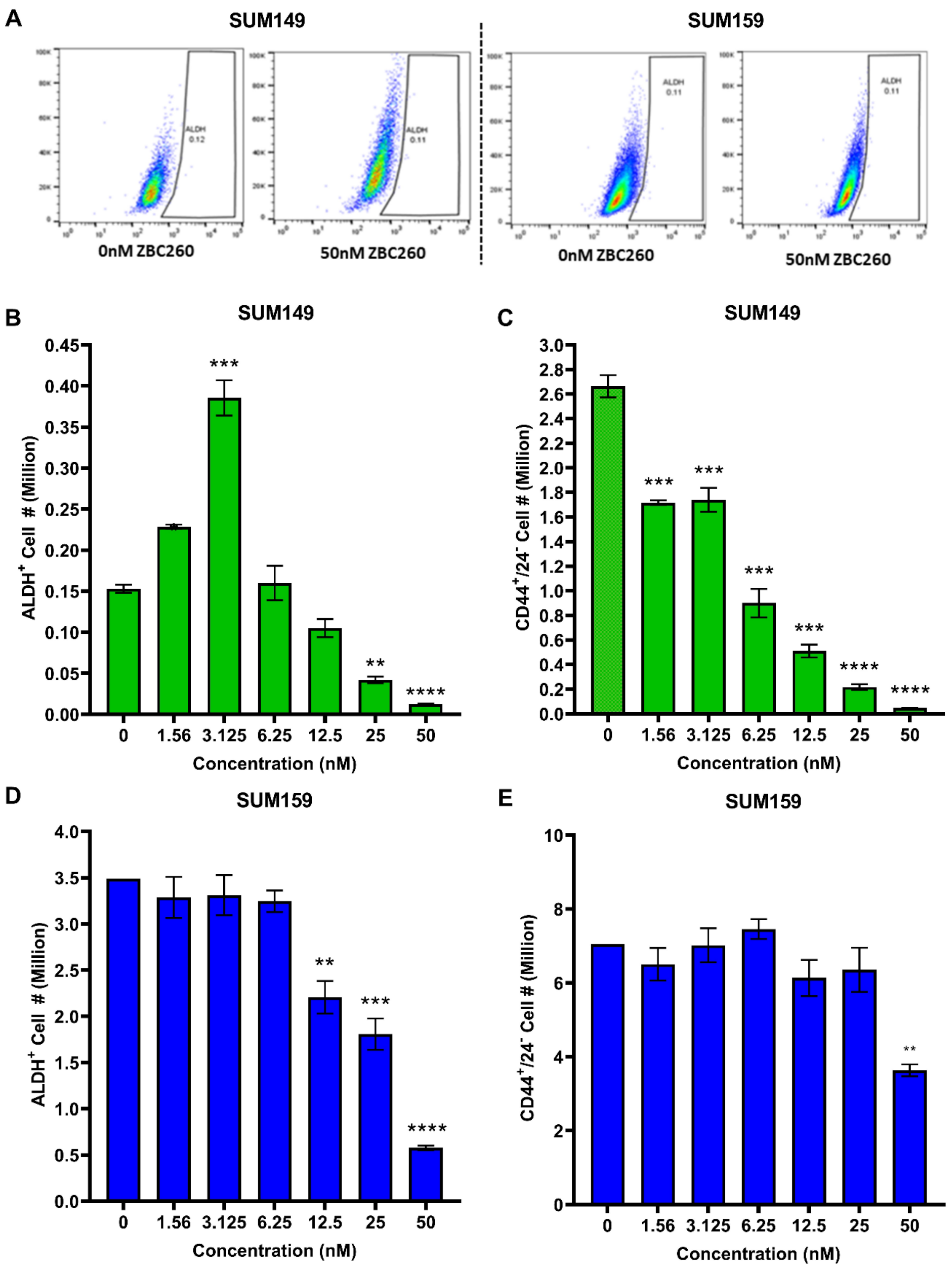

Supplementary Figure 3. Effect of ZBC260 treatment on the absolute number of ALDH$^+$ and CD44$^+$/CD24$^-$ cells. **(a)** Representative flow cytometry picture for the DEAB control for ALDH$^+$ population analysis in SUM149 and SUM159 cells respectively **(b-e)** Concentration-dependent effect of ZBC260 on the absolute number of ALDH$^+$ and CD44$^+$/CD24$^-$ cells in SUM149 cell line **(b, c)** and SUM159 **(d, e)**. Data points are the mean ±SEM for individual experiments, n= 3 independent experiments. The significance level was calculated compared to vehicle control *P< 0.05; ***P* < 0.01; ****P* < 0.001.

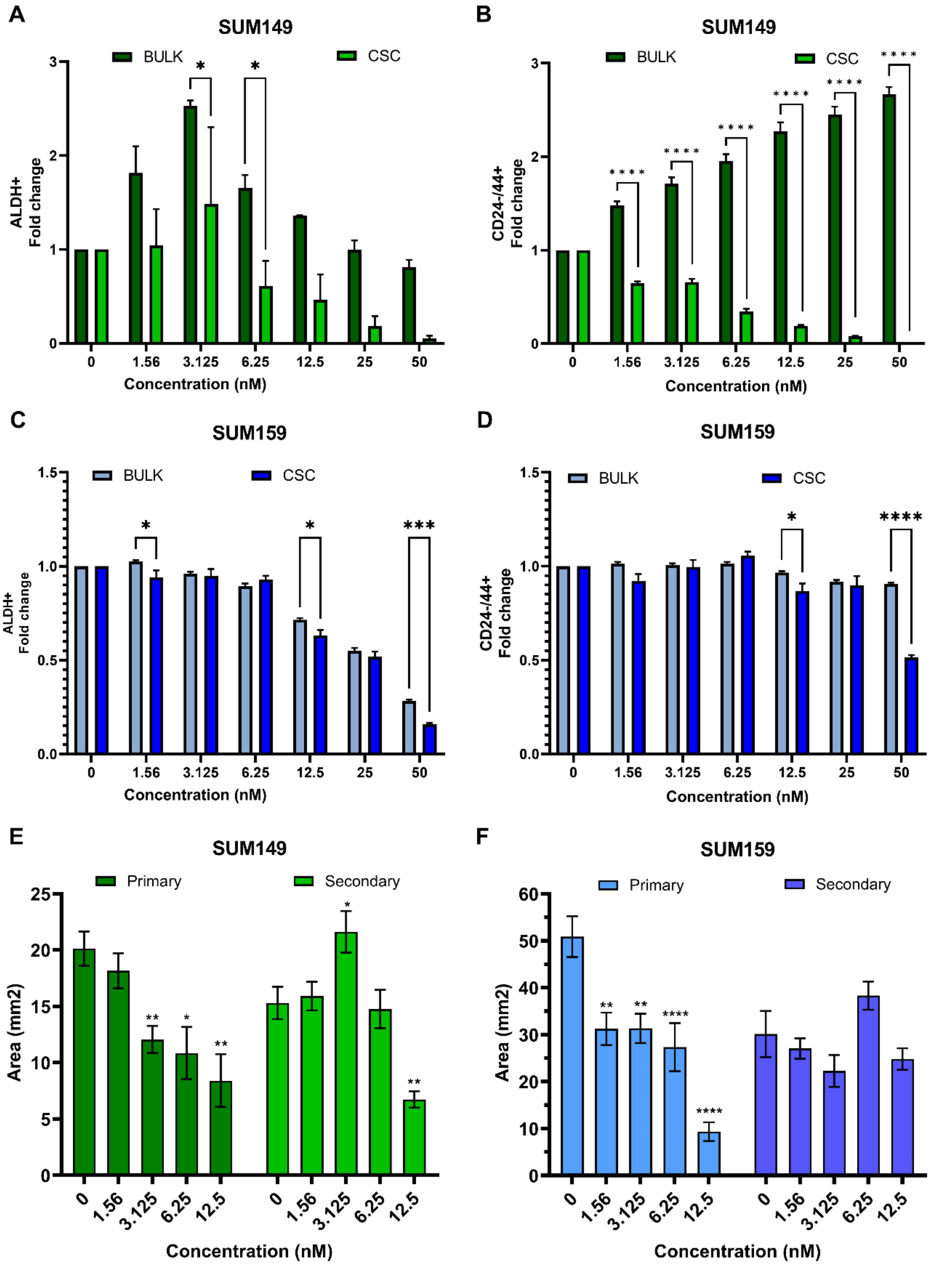

Supplementary Figure 4. Effect of ZBC260 treatment on ALDH^{+} and CD44^{+}/CD24^{-} marker of bulk cells and CSC cells and tumorsphere size

(a-d) Concentration-dependent effect of ZBC260 on the relative ALDH^{+} and CD44^{+}/CD24^{-} cells in SUM149 cell line **(a, b)** and SUM159 **(c, d)**. **(e, f)** Concentration-dependent effect of ZBC260 on primary and secondary tumorsphere size in SUM149 and SUM159 cell lines, respectively. Data points are the mean ±SEM for individual experiments, n= 3 independent experiments. The significance level was calculated compared to vehicle control *P< 0.05; ***P* < 0.01; ****P* < 0.001.

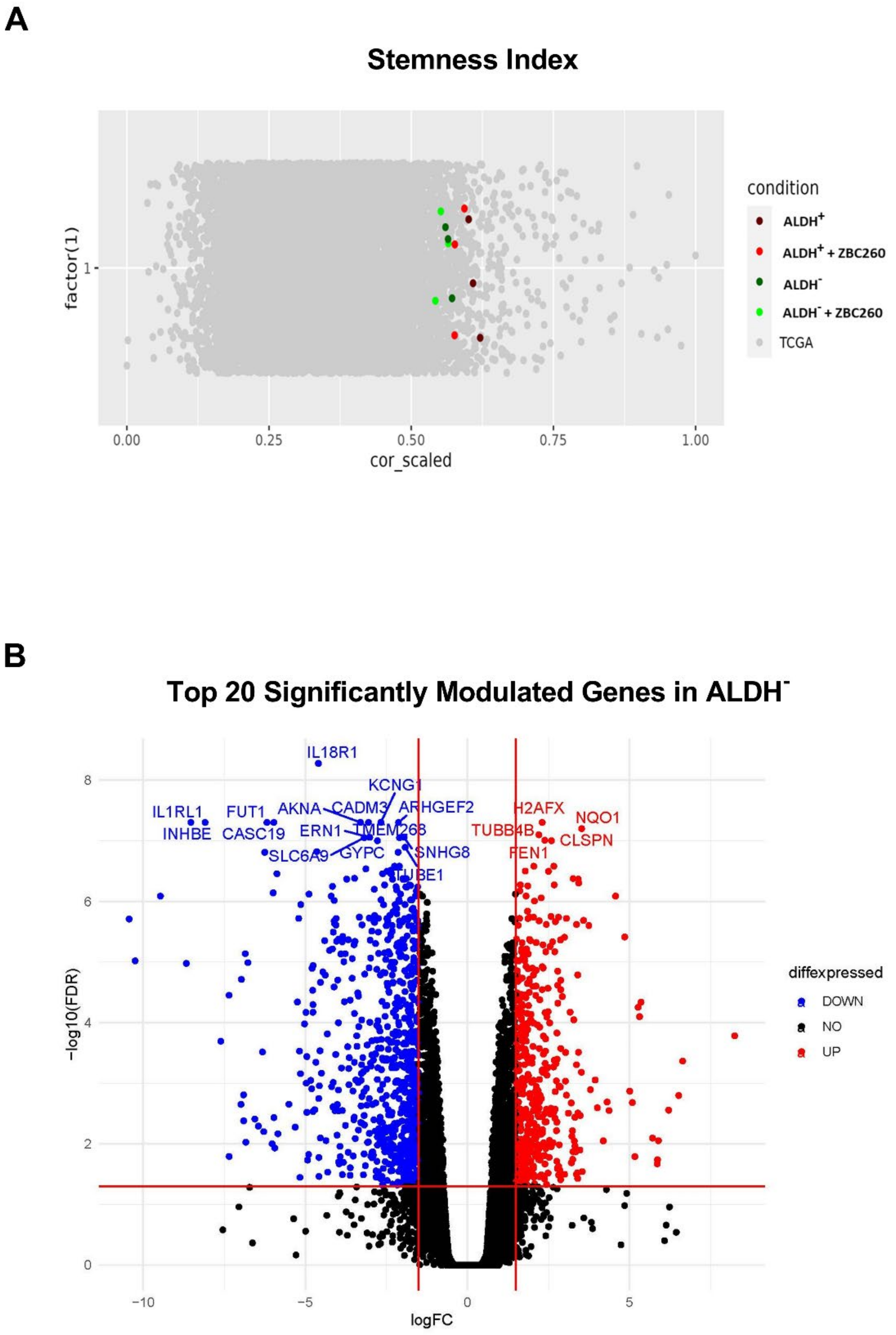

Supplementary Figure 5. Effect of ZBC260 treatment on the transcriptome of SUM159 cells.
(a) Stemness index distribution of SUM159 samples compared to TCGA breast tumors. **(b)** Volcano plot of 20 most significantly expressed genes in SUM159 ALDH$^-$ cell population. Statistics by Benjamini-Hochberg multiple comparisons test, n=3 independent experiments.

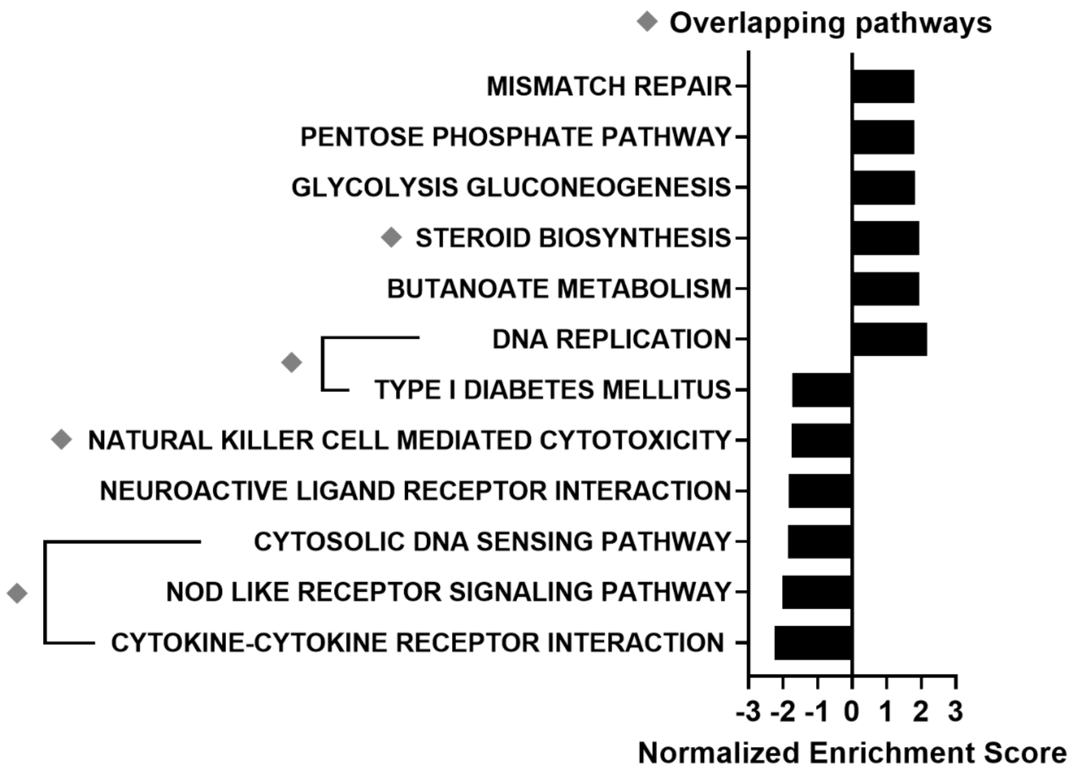

Supplementary Figure 6. Effect of ZBC260 treatment on signaling pathways in ALDH$^-$ cells.
GSEA analysis of the significantly modulated pathways in ALDH$^-$ cells. RNAseq experiment was performed in SUM159 cells and FDR ($P<0.05$) was considered significant. Statistics by Benjamini-Hochberg multiple comparisons test, n=3 independent experiments.

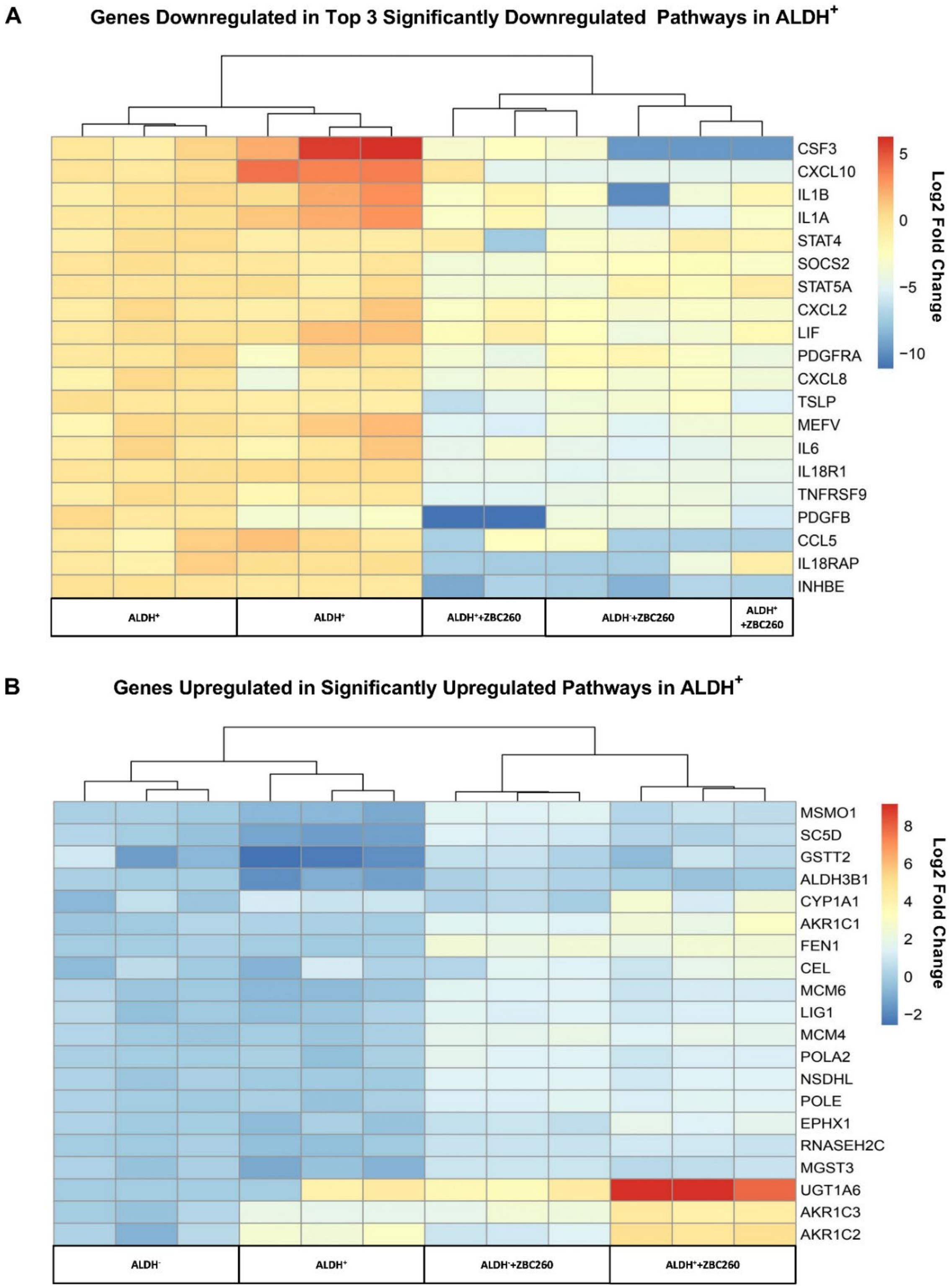

Supplementary Figure 7. Effect of ZBC260 on signaling pathways in ALDH$^+$ cells.
(a) Heat map of 20 most differentially downregulated genes involved in Top3 significantly downregulated pathways in ALDH$^+$ cells. RNAseq experiment was performed in SUM159 cells and FDR (P<0.05) was considered significant. Statistics by Benjamini-Hochberg multiple comparisons test, n=3 independent experiments. **(b)** Heat map of 20 most differentially upregulated genes involved in significantly upregulated pathways in ALDH$^+$ cells.

Tables

Supplementary Table 1. Antibodies used for Western Blotting.

Antibodies	Catalogs and Companies
**BRD2**	Cat#ab264420, ABCAM
**BRD3**	Cat#ab264420, ABCAM
**BRD4**	Cat#A301985A100, BETHYL
**Myc**	Cat#18583S, Cell Signaling Technology
**β-Actin**	Cat#A3854, Sigma-Aldrich
**Stat1**	Cat#14994, Cell Signaling Technology
**pStat1Tyr701**	Cat#7649, Cell Signaling Technology
**Stat3**	Cat#9139, Cell Signaling Technology
**pStat3Tyr705**	Cat#9145S, Cell Signaling Technology
**Stat5**	Cat#D3N2B, Cell Signaling Technology
**pStat5Y694**	Cat#D47E7, Cell Signaling Technology
**Secondary anti-mouse-HRP**	Cat#sc-2005, Santacruz
**Secondary anti-rabbit-HRP**	Cat#7074S, Cell Signaling Technology)

Supplementary Table 2. Human TaqMan Gene Expression Assay primer/probes (Thermo Fisher Scientific) used for real-time quantitative PCR.

Genes	Probe number
GAPDH	Hs02786624_g1
MYC	Hs00153408_m1
CCL5	Hs99999048_m1
CSF3	Hs99999083_m1
CXCL10	Hs00171042_m1
LIF	Hs01055668_m1
PDGFRA	Hs00998018_m1
IL6	Hs00174131_m1
IL18R1	Hs00175381_m1
STAT1	Hs01013996_m1
STAT3	Hs00374280_m1
STAT5A	Hs00559637_g1
