# Supplementary material for: The BET degrader ZBC260 suppresses stemness and tumorigenesis and promotes differentiation in triple-negative breast cancer by disrupting inflammatory signaling
Source: Breast Cancer Res. 2023 Nov 15;25:144. doi: 10.1186/s13058-023-01715-3 (PMC10648675; doi:10.1186/s13058-023-01715-3)
Supplement: Supplementary file 2 — Additional file 2. Uncropped western blots relating to manuscript figures. (a–e) Uncropped western blots for (a) BRD2, (b) BRD3, (c) BRD4, (d) Actin, and (e) MYC; as shown in Figure 1. Lane 1 & 15 – ladder (198-3kDa), lanes 2-7 – SUM149 treated with 0, 1.56, 3.125, 6.25, 12.5, and 25 nM ZBC260 respectively, lane 8 – ladder (460-31kda), and lanes 9-14 – SUM159 treated with 0, 1.56, 3.125, 6.25, 12.5, and 25 nM ZBC260 respectively. (f–l) Uncropped western blots of ALDH− and ALDH+ SUM159 cell treated with ZBC260 (f) Stat1, (g) pStat1, (h) Stat3, (i) pStat3, (j) Stat5, (k) pStat5, and (l) β-actin; as shown in Figure 7. Lane 1 & 15 – ladder (198-3kDa), lanes 2-4 – SUM159 ALDH− + Control, lanes 5-7 – SUM159 ALDH− + ZBC260, lane8 – ladder (460-31kda), lanes 9-11 – SUM159 ALDH+ + Control, lanes 5-7 – SUM159 ALDH+ + ZBC260. [file 13058_2023_1715_MOESM2_ESM.pdf]

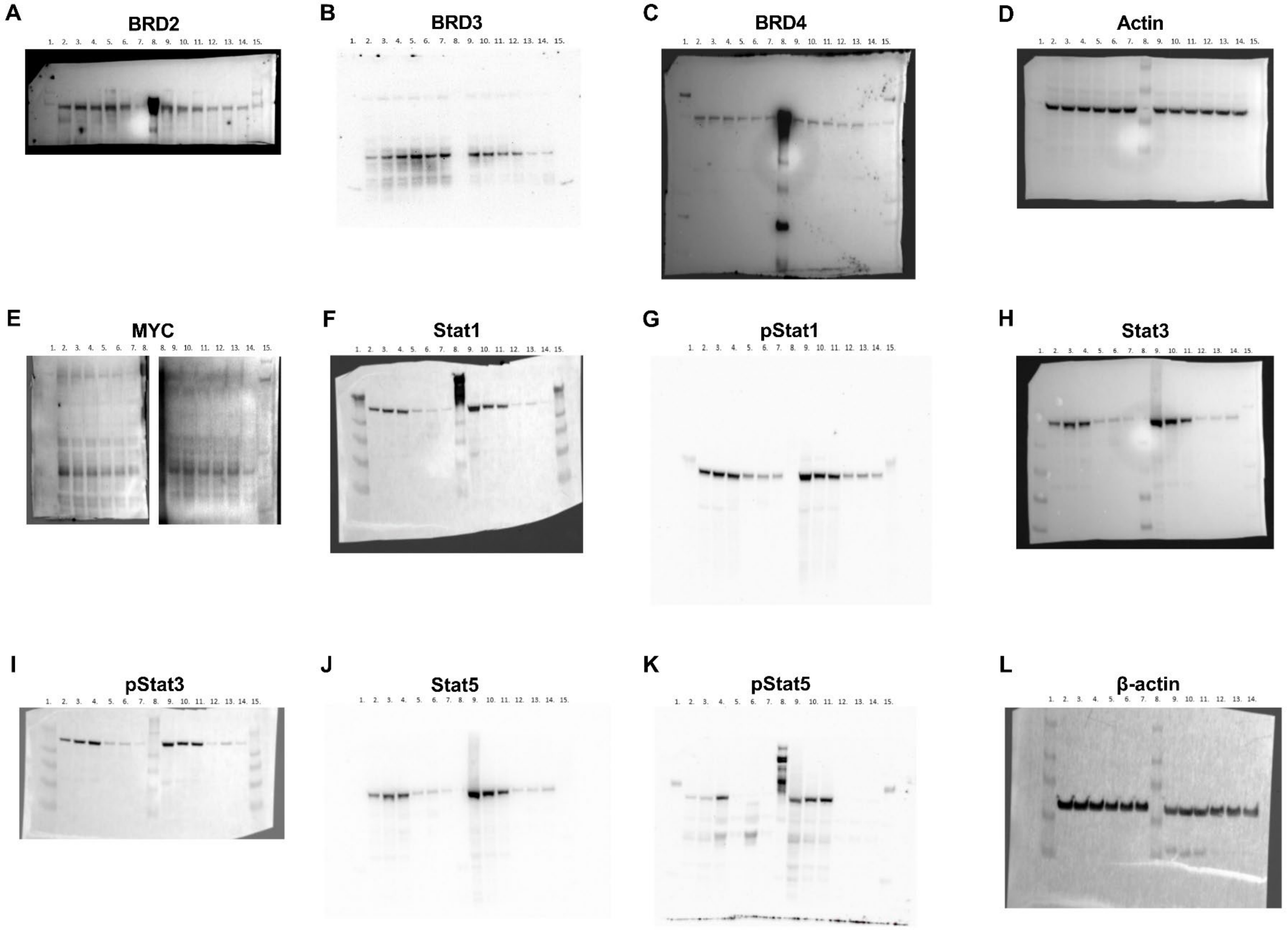

Uncropped western blots relating to manuscript figures.
(a-e) Uncropped western blots for (a) BRD2, (b) BRD3, (c) BRD4, (d) Actin, and (e) MYC; as shown in Figure 1. Lane 1 & 15 – ladder (198-3kDa), lanes 2-7 – SUM149 treated with 0, 1.56, 3.125, 6.25, 12.5, and 25 nM ZBC260 respectively, lane 8 – ladder (460-31kda), and lanes 9-14 – SUM159 treated with 0, 1.56, 3.125, 6.25, 12.5, and 25 nM ZBC260 respectively. **(f-l)** Uncropped western blots of $ALDH^-$ and $ALDH^+$ SUM159 cell treated with ZBC260 (f) Stat1, (g) pStat1, (h) Stat3, (i) pStat3, (j) Stat5, (k) pStat5, and (l) β-actin; as shown in Figure 7. Lane 1 & 15 – ladder (198-3kDa), lanes 2-4 – SUM159 $ALDH^-$ + Control, lanes 5-7 – SUM159 $ALDH^-$ + ZBC260, lane8 – ladder (460-31kda), lanes 9-11 – SUM159 $ALDH^+$ + Control, lanes 5-7 – SUM159 $ALDH^+$ + ZBC260.
